# Distinct but interchangeable subpopulations of colorectal cancer cells with different growth fates and drug sensitivity

**DOI:** 10.1016/j.isci.2023.105962

**Published:** 2023-01-13

**Authors:** Roberto Coppo, Jumpei Kondo, Keita Iida, Mariko Okada, Kunishige Onuma, Yoshihisa Tanaka, Mayumi Kamada, Masayuki Ohue, Kenji Kawada, Kazutaka Obama, Masahiro Inoue

**Affiliations:** 1 Department of Clinical Bio-resource Research and Development, Graduate School of Medicine, Kyoto University, Kyoto, Japan; 2 Institute for Protein Research, Osaka University, Suita, Osaka, Japan; 3 Graduate School of Pharmaceutical Sciences, Kyoto University, Kyoto, Japan; 4 RIKEN Center for Computational Science, HPC- and AI-driven Drug Development Platform Division, Biomedical Computational Intelligence Unit, Hyogo, Japan; 5 Department of Biomedical Data Intelligence, Graduate School of Medicine, Kyoto University, Kyoto, Japan; 6 Department of Gastroenterological Surgery, Osaka International Cancer Institute, Osaka, Japan; 7 Department of Surgery, Graduate School of Medicine, Kyoto University, Kyoto, Japan

**Keywords:** Therapy, Cell biology, Cancer

## Abstract

Dynamic changes in cell properties lead to intratumor heterogeneity; however, the mechanisms of nongenetic cellular plasticity remain elusive. When the fate of each cell from colorectal cancer organoids was tracked through a clonogenic growth assay, the cells showed a wide range of growth ability even within the clonal organoids, consisting of distinct subpopulations; the cells generating large spheroids and the cells generating small spheroids. The cells from the small spheroids generated only small spheroids (S-pattern), while the cells from the large spheroids generated both small and large spheroids (D-pattern), both of which were tumorigenic. Transition from the S-pattern to the D-pattern occurred by various extrinsic triggers, in which Notch signaling and Musashi-1 played a key role. The S-pattern spheroids were resistant to chemotherapy and transited to the D-pattern upon drug treatment through Notch signaling. As the transition is linked to the drug resistance, it can be a therapeutic target.

## Highlights

• Subpopulations with different growth ability and drug sensitivity exist in CRCs
• The cell fate of each cell is distinct but interchangeable
• Notch and Musashi-1 modulate the nongenetic cellular plasticity of CRCs
• Transition of the cell fates occurs upon drug treatment through Notch signaling

## Graphical abstract

**Figure:**
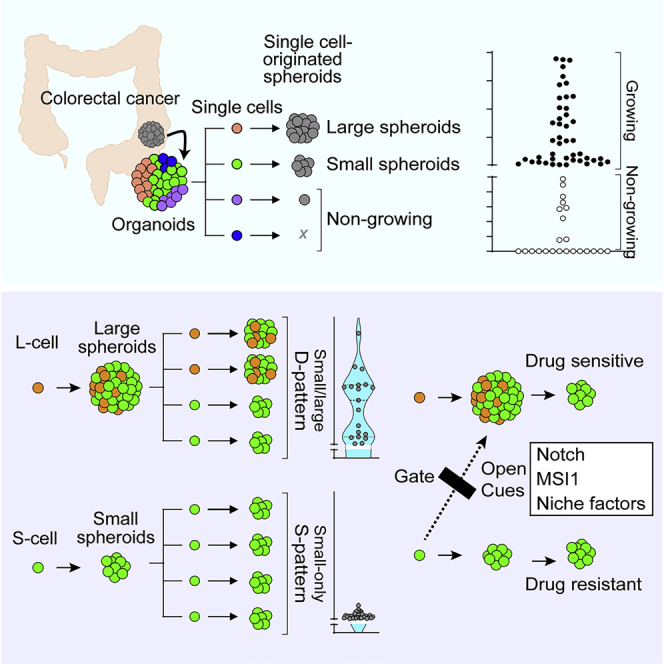

## Introduction

Cancer is characterized by extensive intratumor heterogeneity (ITH).^1^^,^^2^ It has been increasingly recognized that ITH contributes to drug resistance and cancer recurrence following therapy.^3^ ITH is known to be caused by a variety of genetic mutations, microenvironmental conditions, and cell-intrinsic plasticity.^2^^,^^4^^,^^5^ Genetic mutations-driven ITH has been systematically studied,^6^ whereas the ITH-related nongenetic processes remain largely elusive and are currently receiving intense research attention.^7^^,^^8^^,^^9^

Cancer stem-like cells (CSCs) are one of the theories that attempt to explain the nongenetic heterogeneity of cancer.^5^^,^^10^ Recently, CSC models have been revisited with the evidence that cancer cells can dynamically fluctuate from a non-stem cell-like state to a stem cell-like state.^10^ For this reason, the importance of the heterogeneity and plasticity within cancer cell subpopulation is highlighted.^5^^,^^8^

Colorectal cancer (CRC) is one of the leading causes of cancer-related death worldwide.^11^ Accumulating evidence suggests that CRC cells represent phenotypically dynamic (rather than static), heterogeneous cell populations that display cell plasticity characteristics.^12^^,^^13^^,^^14^^,^^15^^,^^16^ Recently, 3D cell culture systems utilizing patient-derived tumors have been developed for various cancer types, including CRC.^17^ Herein, we used the cancer tissue-originated spheroid (CTOS) method developed by us, in which the cell-cell contact is maintained throughout the organoid preparation, culture, and passaging.^18^ The growth of each CRC organoid within the same line is quite heterogeneous,^19^ suggesting that CRC organoids prepared by CTOS method retain heterogeneous populations of cells. Furthermore, we demonstrated that a small subset of cells within the CRC organoids can initiate regrowth after exposure to high-dose radiation, an observation that was nongenetically and reversibly determined.^20^

As current cancer therapies have been designed and developed mainly against the fast-growing cancer cells, the importance of the quiescent or slow-growing cancer cells has been overlooked. Consequently, these slow-growing cancer cells may survive the anticancer treatment, revert to the fast-growing cancer cells, and serve as a reservoir for tumor regrowth.^13^^,^^21^^,^^22^^,^^23^ To characterize the dynamics of quiescent or slow-growing cancer cells, an analysis at the single-cell resolution is required because the nature of the slow-growing cells is usually masked by that of the fast-growing cells. Recently, single-cell transcriptome analyses were recruited and served the study of the characteristics of the slow-growing CSCs.^24^ However, such a “snapshot” analysis involving a procedure for isolating CSCs with the use of markers^24^ or dye retention^25^ exerts limitations when applied to a continuously changing process. Therefore, the employment of a phenotypically trackable cell culture system allowing for a single-cell resolution is necessary.

In this study, we modified the conventional spheroid-forming assay, with which we tracked the cell fate of forming spheroids as well as the growth at a single-cell resolution and revealed the existence of heterogeneous subpopulations in CRC organoids with distinct growth patterns. We characterized the distinct but interchangeable subpopulations, and revealed the molecular mechanisms regulating the transition.

## Results

### Heterogeneous growth ability of the cells in CRC organoids at a single-cell level

**Figure 1:**
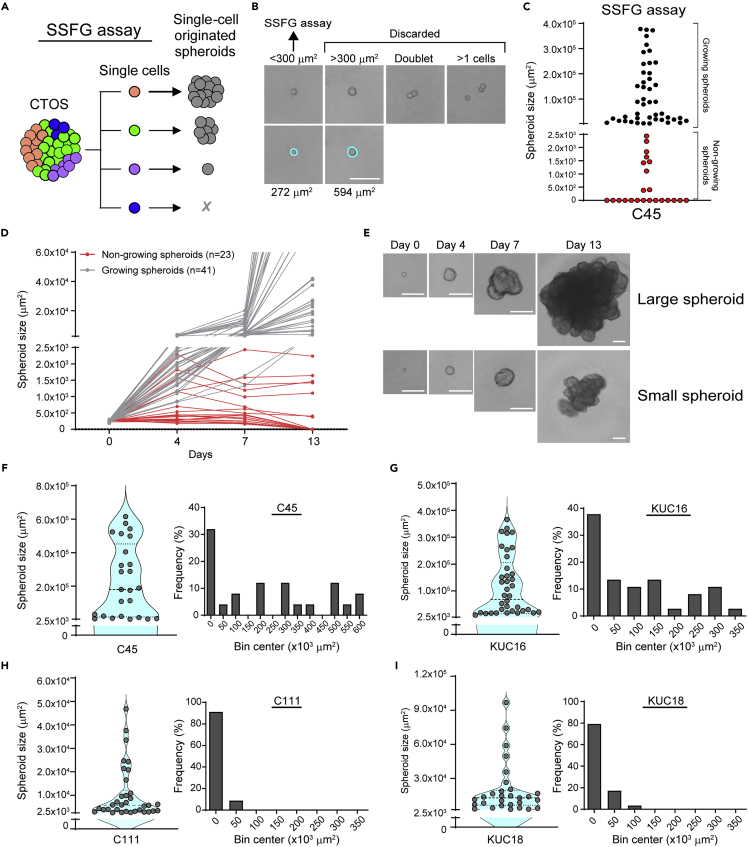
CRC organoids exhibit heterogeneous growth at the single-cell level (A) Schematic overview of the SSFG assay undertaken. A circle represents a cell; different colors represent heterogeneous populations of the cells. (B) Phase-contrast images of the SSFG assay at day 0. The blue circles indicate the projected area of single cells. (C) Size distribution of the C45 single-cell-derived spheroids in the SSFG assay. Black and red dots represent growing and non-growing spheroids, respectively. (D) Growth curve of the growing (gray) and the non-growing (red) spheroids from (1C). (E) Phase-contrast images of the representative growing spheroids in (1C). (F–I) Left panel: a violin plot of the SSFG assay. Right panel: frequency distribution of the size of the growing spheroids. All scale bars in Figure 1: 100 μm.

To precisely track the cell fate of the growth in CRC organoids at a single-cell resolution, we modified the conventional spheroid-forming assay and developed a single-cell-derived spheroid-forming and growth (SSFG) assay, which includes the undertaking of (i) an initial confirmation of the strict single-cell status within a well, (ii) culture under growth-permissive conditions, and (iii) a time-course growth assessment of each well (Figure 1A). We applied the SSFG assay to a CRC organoid line, C45, which has wide range of spheroid growth abilities.^19^ We excluded non-single cells and large cells which can be doublets or the cells just before cell division at the very beginning of the assay (Figure 1B), as well as the non-growing cells (Figures 1C and 1D) that presented with several patterns: early death (Figure S1A-a), late death (Figures S1A-b ), growth arrest (Figures S1A-c), and a decline in spheroid size (Figures S1A-d). The maximum area of the non-growing spheroids was below 2.5 × 10^3^ μm^2^. The spheroid-forming capacity was, on average, 59%. The growth range of the single-cell-derived growing spheroids in the C45 line was 228-fold (Figures 1C–1F). We measured the growth variation of the spheroids derived from single cells through the SSFG assay in 13 additional lines of CRC organoids from different patient tumors (Table S1, Figures 1G–1I, and S1B–S1K). The spheroid-forming capacities were between 19% and 59% (Table S2). In all 14 lines (including C45), the sizes of the spheroids within each line varied substantially, and two of the lines (C120 and C132) demonstrated a statistically significant bimodal distribution. The mutational profile (Table S3) showed no clear correlation with the spheroid-forming capacity, the growth, or the growth range. These results indicate that each spheroid-forming cell in the CRC organoids has diversity of the growth ability as a single cell.

### Each clone from the CRC organoids generated distinct subpopulations with different growth capacity

**Figure 2:**
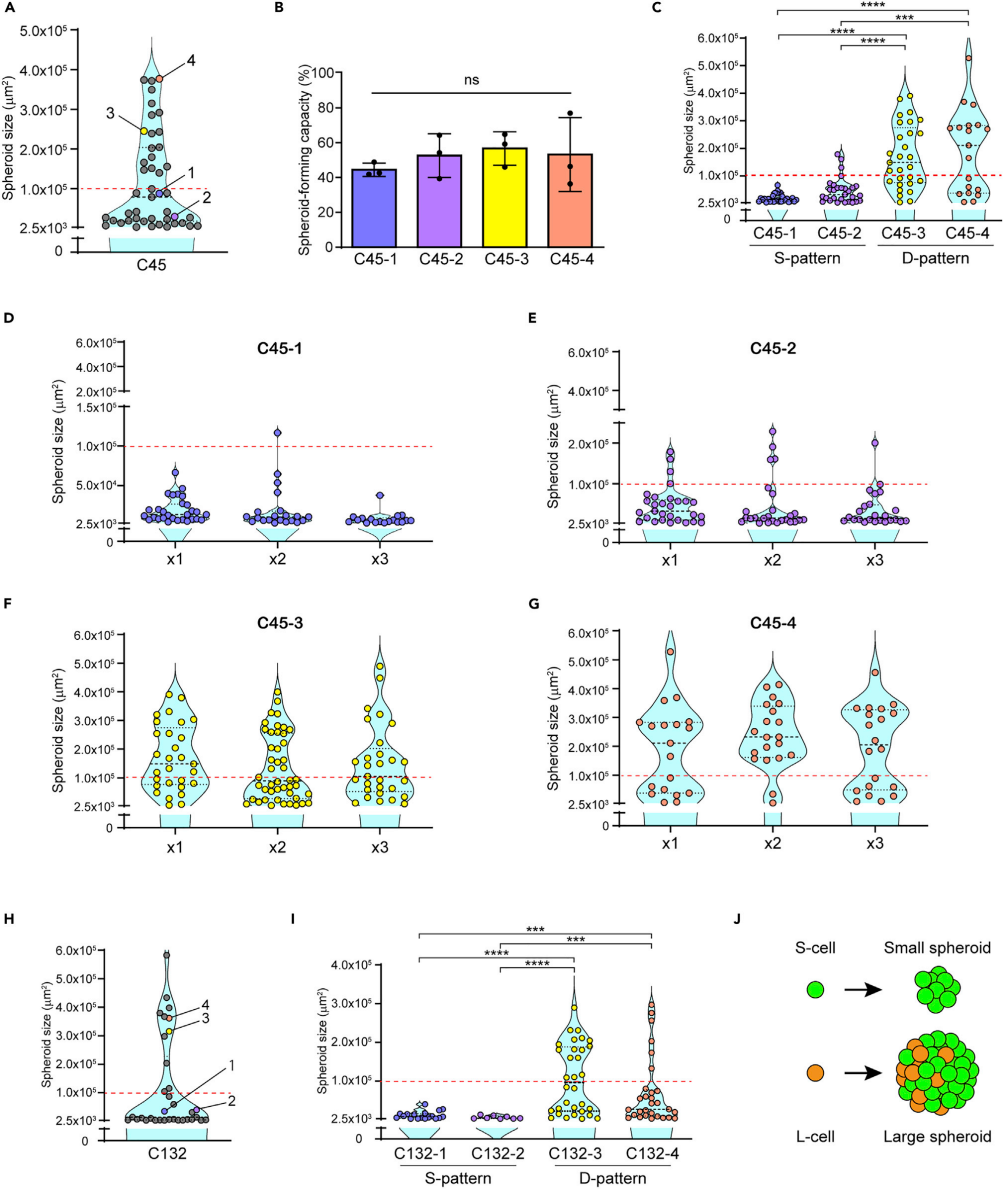
Each clone from the CRC organoids generated distinct subpopulations with different capacity of growth (A) Violin plots of the SSFG assay for the C45 CRC organoid line. Four selected spheroids are indicated. The red dashed line indicates a rounded value (1.0 × 10^5^ μm^2^) of the area at day 13, for the C45-1 line, and it is indicative of a putative threshold between the small and the large spheroids in the C45 lines. (B) Spheroid-forming capacity of the indicated clones. The mean ± SD is shown, tested by one-way ANOVA, followed by Tukey’s test. (C) Violin plots of the SSFG assay for the indicated clones. (D–G) Violin plots of the serial SSFG assays for the indicated clones. The first round is indicated as “x1,” the second as “x2,” and the third as “x3.” The results of the first round are the same as in (2C). (H) Violin plots of the SSFG assay for the C132 line. Four selected clones are highlighted. (I) Violin plots of the SSFG assay for the indicated C132 clones. (J) Schematic overview of the growth characteristics of a single cell. A circle represents a cell; green, S-cells; orange, L-cells. Note that the component of the spheroids is assessable only after the next round SSFG assay. In Figure 2, the data of the SSFG assay were tested by the Mann–Whitney U test. ∗∗∗, p < 0.001; and ∗∗∗∗, p < 0.0001.

To characterize the heterogeneous capacity of spheroid formation and growth of the single cells in more detail, we selected several individual spheroids at the end of the first round of SSFG assay using C45 line (Figure 2A). The growth curves and the images of each clone in the first round SSFG assay are shown in Figures S2A and S2B. As each spheroid was strictly derived from a single cell, it can be called a clone. We named the clones as C45-1 and C45-2 from the small spheroids and two clones as C45-3 and C45-4 from the large spheroids. To characterize each clone, we first evaluated its proliferation capacity and viability. To evaluate proliferation, we immunostained PCNA, a proliferating cell nuclear antigen. The PCNA-positive rates were higher in the large spheroids (C45-3 and C45-4) compared to the small spheroids (C45-1 and C45-2) (Figures S3A and S3B), suggesting a higher proliferative state of the cells in the large spheroid-forming clones during *in vitro* culture. To evaluate cell death, we performed propidium iodide (PI) staining, which showed no differences in viability between the C45 clones during culture (Figures S3C and S3D). Consistent with these results, all the C45 clones had similar spheroid-forming capacity (Figure 2B), suggesting that the differing growth capacity was due to the differences in the cell proliferation rather than survival. Next, we expanded each clone *in vitro* and performed the second round of SSFG assays. The spheroid-forming capacity was preserved in all clones (Figure 2B). The cells derived from the small spheroids gave rise to only small spheroids (S (small)-pattern), while the cells derived from the large spheroids gave rise to both small and large spheroids (D (dual)-pattern) (Figure 2C). We then set the putative threshold between the two phenotypes at 1.0 × 10^5^ μm^2^, based on the rounded value of the maximum size of the C45-1 spheroids at day 13. To further investigate the stability of the growth features, we performed additional rounds of the SSFG assay. In all four C45 clones, the growth pattern of single cells was preserved during all three rounds of the SSFG assay (Figures 2D–2G). We could confirm the distinct growth patterns of single cells in three other CRC organoid lines (Figures 2H, 2I, and S2C–S2F). These results indicate that the spheroid-forming cells within each CRC organoid consisted of two different types of cells; a cell which has the capacity of generating a small spheroid, an S-cell, and generating a large spheroid, an L-cell. During spheroid formation, an S-cell only gave rise to a small spheroid consisting of pure S-cells, while an L-cell gave rise to a large spheroid consisting of both the S- and the L-cells (Figure 2J).

### Establishment of an S-cell clone from the small spheroids derived from an L-cell clone

**Figure 3:**
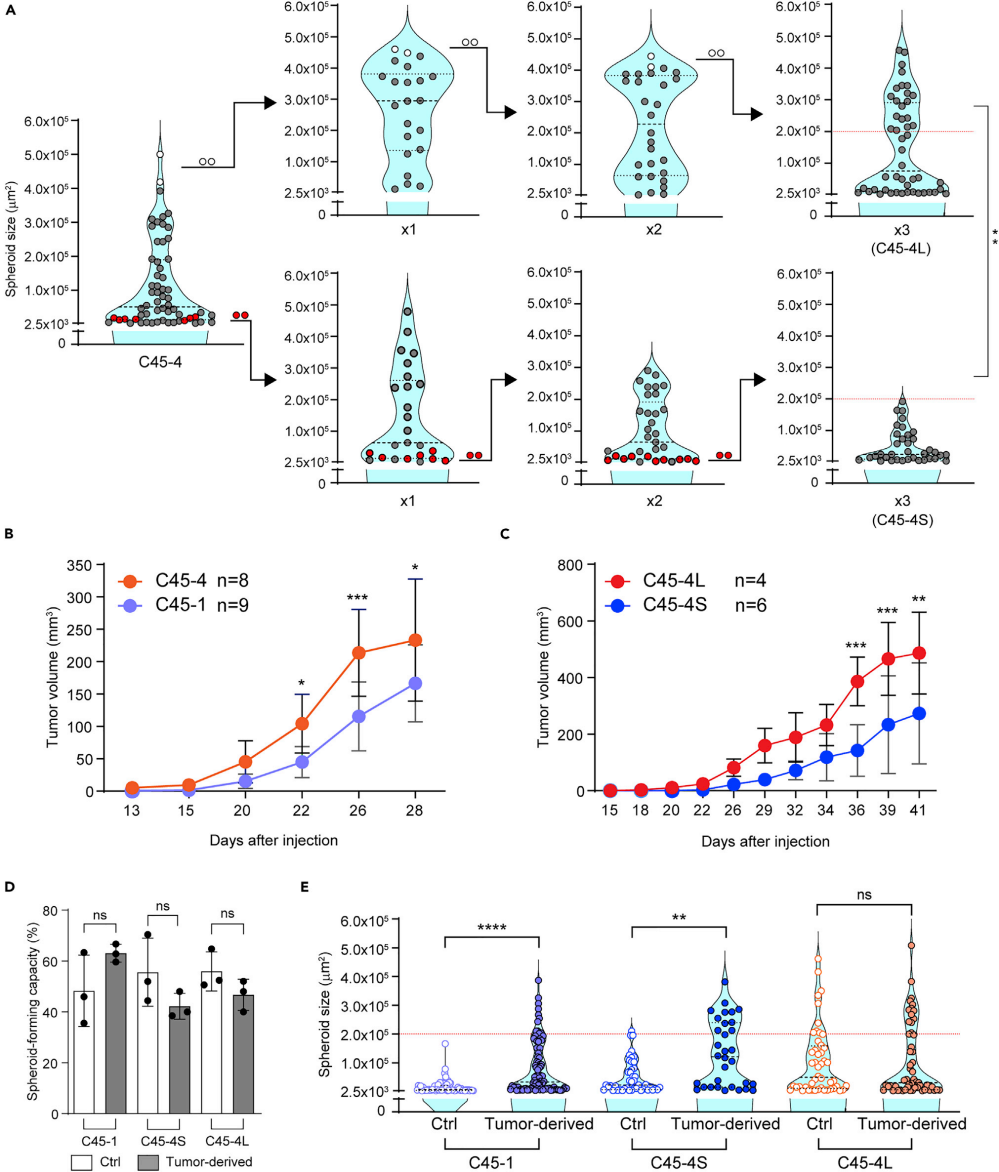
Distinct but interchangeable features of L-cells and S-cells (A) Violin plots of the SSFG assay for C45-4 clones. Red and open circles represent the pooled small and large spheroids, respectively. Several large and small spheroids with similar size were collected separately and subjected to the next round of the SSFG assay. Three rounds of the SSFG assay were performed for each subclone. The first round is indicated as “x1,” the second as “x2,” and the third as “x3” (C45-4L, -4S). The red dotted line expresses the rounded value (2.0 × 10^5^ μm^2^) of the area at day 13, for the C45-4S line, and it is indicative of a putative threshold between the small and the large spheroids in the C45-4 lines. (B and C) Growth curves of xenograft tumors originating from C45-1 and -4 (3B) and C45-4L and -4S (3C) spheroids. The mean ± SD is shown; n, the number of animals in each group; statistical analyses were performed by two-way ANOVA, followed by Bonferroni’s test. N, number of mice. (D and E) Spheroid-forming capacity (3D) and violin plots of the SSFG assay (3E) for the C45-1, C45-4S, and C45-4L subclones comparing control organoids maintained *in vitro* (Ctrl) with those prepared from the xenografts in (3B, 3C) (tumor-derived). In Figure 3, the data of the spheroid-forming capacity were tested by one-way ANOVA, followed by Tukey’s test, and those of the SSFG assay were tested by the Mann–Whitney U test. Ns, not statistically significant. ∗, p < 0.05; ∗∗, p < 0.01; ∗∗∗, p < 0.001; and ∗∗∗∗, p < 0.0001.

Next, we investigated the fate of the cells in the small spheroids of the L-cell clone, C45-4. From the first round of SSFG assay, we collected small and large spheroids, respectively (Figure 3A). We then repeated the SSFG assay followed by the collection three times (Figure 3A). The D-pattern was stably preserved in the large-spheroids pool. However, the small-spheroids pool exhibited the D-pattern in the first round, and then the ratio of the large spheroids decreased at the second round and showed the S-pattern in the third round, the phenotype of which was stable in the subsequent experiments. We then named the subclones of C45-4 after three rounds of the SSFG assay derived from the small spheroids and the large spheroids as C45-4S and C45-4L, respectively. The spheroid-forming capacity of both subgroups did not differ from that of the parent C45-4 (see Figure 2C and the control groups in the following results of the SSFG assay). The disappearance of the L-cells in the small-spheroids pool during the multiple rounds of SSFG assay might be attributable to an epigenetic event, while it can also be caused by the elimination of the contaminated L-cells in the small spheroids. In any way, stable phenotype of the S-cells could be generated from the L-cells. The ratio of PCNA-positive cells was higher in C45-4L spheroids compared to the C45-4S spheroids, suggesting more proliferation of the cells in the large spheroid subclone (C45-4L) (Figures S4A and S4B). In contrast, there were no differences in PI staining between the C45-4L and C45-4S subclones (Figures S4C and S4D). Since the C45-4 spheroids were derived from a single clone, the growth pattern of single cells is likely to be regulated by nongenetic mechanisms.

### The L-cells can be generated from the S-cells *in vivo*

We next examined the tumorigenicity of the clones. We subcutaneously injected the spheroids of the S-cell clones, C45-1 and C45-4S, and the L-cell clones, C45-4 and C45-4L, into immunodeficient mice. All spheroids were tumorigenic (Figures 3B and 3C). Although the S-clones demonstrated a latency period of tumor growth, which was not observed in the L-clones, S-clones’ growth rate eventually caught up. To confirm initially that S-cells were capable of accelerating the cell proliferation under *in vivo* conditions, we examined the tumor xenografts at two different time points: a “midpoint”, when the tumor volume was detectable for the first time, and an “endpoint”, when the tumor volume reached ∼200 mm^3^. PCNA-positive rate increased from 30% *in vitro* to an average of 50%–60% in the xenograft tumors at the midpoint and endpoint, reaching the levels of D-pattern spheroids (Figure S5). This suggests that S-cells are capable of restarting the cell cycle *in vivo*. The change in the proliferation rate was already observed at the beginning of the tumor growth, indicating that the transition of S-cells to a D-pattern phenotype occurred during the latency period.

In contrast, the PCNA-positive rates of D-pattern spheroid-derived xenograft tumors were the same in the *in vitro* and *in vivo* conditions (Figure S5). These results suggested that S-cells and L-cells have different dependencies on the tumor microenvironment for regulating proliferation. We then prepared organoids from the xenografts and subjected them to the SSFG assay. The spheroid-forming capacity was preserved after the xenograft formation (Figure 3D). The D-pattern was preserved in the cells of C45-4L spheroids (Figure 3E). Interestingly, the S-cell clones, C45-1 and C45-4S, acquired the D-pattern phenotype following the xenograft formation (Figure 3E). These results indicate that the L-cells can be generated from the S-cells after interacting with the tumor microenvironment, although we could not exclude the possibility that the phenotype change was attributable to the contamination of the small number of the L-cells in the S-cell clones, even if they were undetectable through the multiple rounds of the SSFG assay *in vitro*.

### Different gene expression profiles of the spheroids with the different growth patterns

**Figure 4:**
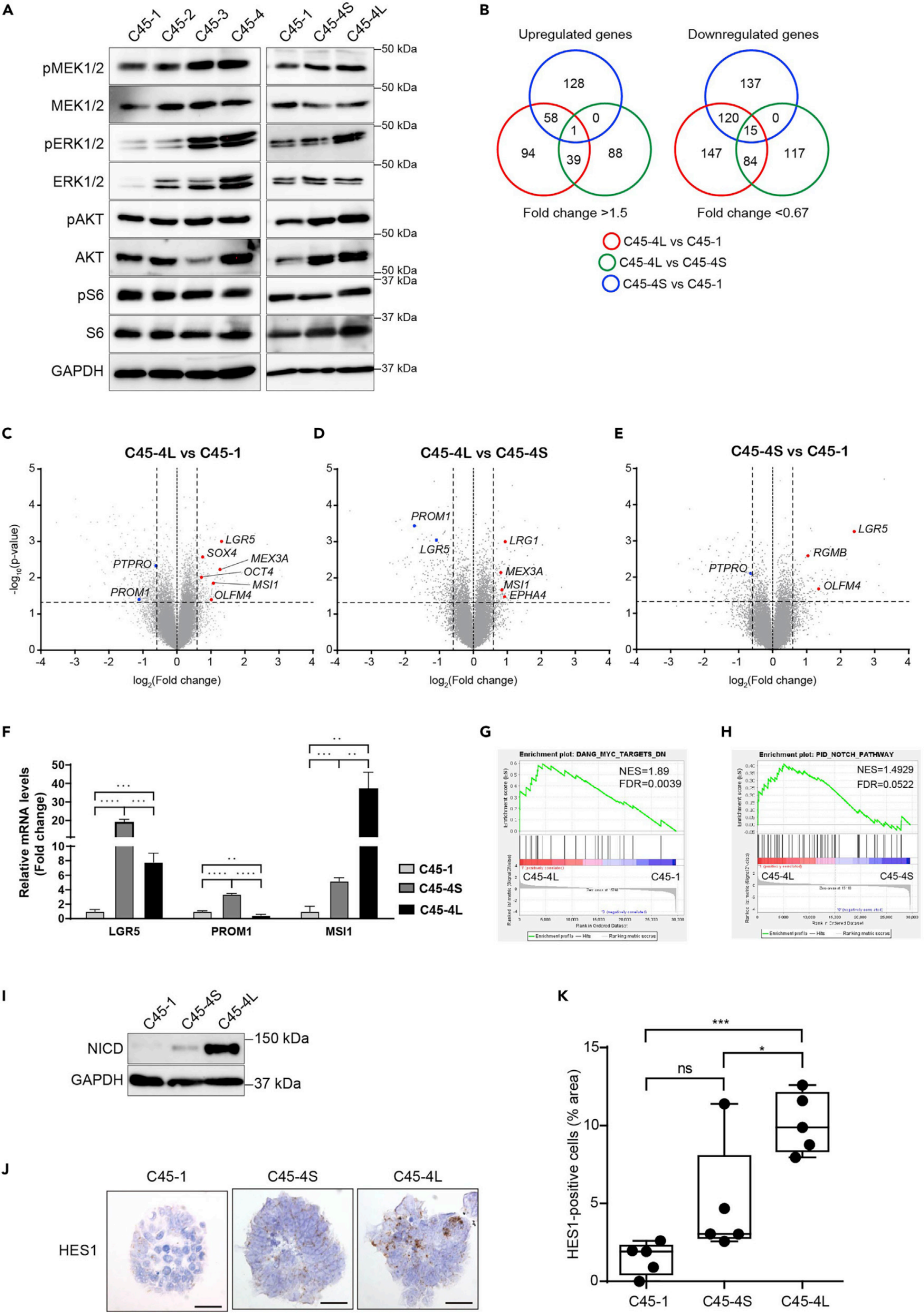
Transcriptome analysis revealed a heterogeneous expression of stemness-related genes among the CRC spheroids (A) Western blots analysis of the intracellular signaling in the spheroids of the indicated clones after 7 days of culture. (B) Venn diagram presenting the number of significantly upregulated (left panel) and downregulated (right panel) genes in the comparison of C45-1, C45-4S, and C45-4L spheroids. (C–E) Volcano plots displaying the differentially expressed genes from gene expression microarray data obtained from three independent experiments (n = 3). (4C) C45-4L vs. C45-1, (4D) C45-4L vs. C45-4S, and (4E) C45-4S vs. C45-1. The red and blue dots represent the upregulated and downregulated colorectal CSC marker genes, respectively. The threshold lines are indicated as ± 0.59 for the log2 (fold change) and as 1.3 for the − log10 (p value). (F) Relative mRNA levels of the *LGR5*, *PROM1* (CD133), and *MSI1* genes in the indicated C45 clones after 7 days of culturing. (G and H) Gene set enrichment analysis (GSEA) of the transcriptome data by using (G) the c-Myc target gene signature (DANG_MYC_TARGETS_DN) or (H) the Notch pathway activation (PID_NOTCH_PATHWAY), and comparing the D-pattern C45-4L with the S-pattern C45-1 and C45-4S, respectively. (I) Western blot analysis of the expression of NICD in the indicated spheroids. (J) Representative images of HES1 *in situ* hybridization in the indicated spheroids. Scale bars: 50 μm. (K) Quantitative analysis of (J). The area of HES1-positive cells was calculated, normalized per total nuclei area, and is shown as a percentage. In (F) and (K), the data were tested by one-way ANOVA, followed by Tukey’s test. Ns, not statistically significant. ∗, p < 0.05; ∗∗, p < 0.01; ∗∗∗, p < 0.001; and ∗∗∗∗, p < 0.0001.

We examined the differences in the molecular features between the spheroids with the different growth patterns. Direct comparison of the S-cells and the L-cells is impossible because the growth capacity is assessed only after the SSFG assay. Taking advantage of the finding that the spheroids derived from the S-cell clones consisted of the pure S-cells, we compared the S-pattern spheroids derived from the S-cell clones with the D-pattern spheroids derived from the L-cell clones, which consisted of the mixture of the S-cells and the L-cells. As expected from the slow growing feature, the S-pattern spheroids showed less ERK activity than those of the D-pattern spheroids (Figure 4A).

To shed more light on the molecular characteristics, we analyzed the differentially expressed genes between the spheroids with different growth patterns derived from a clone; C45-1, C45-4S, and C45-4L. The single cells from each subclone were cultured for 7 days and were subsequently subjected to microarray analyses. Of the 29,596 genes examined, 408 genes were found to be upregulated more than 1.5-fold, and 620 genes were downregulated less than 0.67-fold when the three aforementioned subclones were compared (Figure 4B and Table S4). The volcano plot analyses revealed the similarity of their gene expression profiles and the existence of some differentially expressed genes (Figures 4C–4E). Surprisingly, among these differentially expressed genes, many have been previously reported as stem cell markers of CRC.^10^^,^^15^^,^^26^ The levels of MSI1, MEX3A, SOX4, EPHA4, and LRIG1 were higher in the C45-4L spheroids; the level of PTPPRO was higher in the C45-1 spheroids; and the levels of LGR5, PROM1 (CD133), and RGMB were higher in the C45-4S spheroids. We could confirm the expression patterns of LGR5, PROM1, and MSI1 by a semi-quantitative RT-PCR (Figure 4F). The results suggested that the reported CSC genes were differentially expressed among these clones. Gene set enrichment analysis (GSEA) revealed that the MYC signature^27^ was significantly enriched in the C45-4L spheroids compared with the C45-1 spheroids (Figure 4G), thereby supporting the growth difference as MYC lies at the crossroads of many growth-promoting signal transduction pathways.^28^ Additionally, GSEA revealed that the Notch pathway activation (PID_NOTCH_PATHWAY)^29^ was enriched in C45-4L spheroids when compared to the C45-4S spheroids (Figure 4H), suggesting the role of Notch signaling in regulating the D-pattern phenotype. Indeed, the protein levels of the Notch intracellular domain (NICD) were higher in the D-pattern spheroids (C45-4L) when compared with the S-pattern spheroids (C45-4S) (Figure 4I). Moreover, we have analyzed the expression of *HES1*, a downstream target of Notch signaling by *in situ* hybridization (RNA-Scope) (Figures 4J and 4K). The expression of *HES1* mRNA in the D-pattern spheroids was higher than that in the S-pattern spheroids. Interestingly, the HES1-positive cells were not homogeneously distributed but were scattered in some small areas. Taken together, the activation of the Notch pathway was higher in the D-pattern spheroids than the S-pattern spheroids.

### Transition between the growth patterns was regulated by cell-cell contact through notch signaling

**Figure 5:**
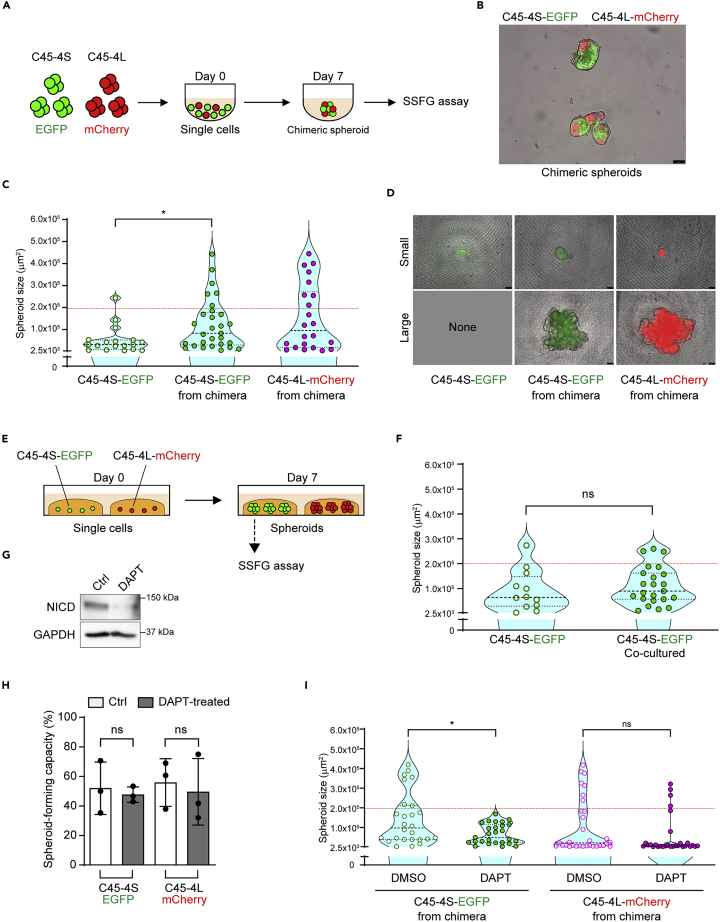
Transition from the S-cells to the L-cells was regulated by cell-cell contact through Notch signaling (A) Schematic overview of the chimeric spheroid experiments that enabled physical cell-cell interactions between cells of different nature; mCherry-labeled C45-4L (red circles) and EGFP-labeled C45-4S (green circles) cells. (B) Representative image of the C45-4L-mCherry/C45-4S-EGFP chimeric spheroids. Scale bar: 75 μm. (C) Violin plots of the SSFG assay comparing the cells from pure C45-4S-EGFP (Ctrl), the EGFP-positive cells from the C45-4L-mCherry/C45-4S-EGFP chimera (Chimera), and the mCherry-positive cells from the chimera. (D) Images of the small and large spheroids, at day 13, derived from EGFP- or mCherry-positive cells. (E) Schematic overview of the co-culture system without cell-cell interactions; mCherry-labeled C45-4L cells, red; EGFP-labeled C45-4S cells, green. (F) Violin plots of the SSFG assay comparing the EGFP-positive cells from the pure C45-4S-EGFP spheroids (Ctrl) with those from the co-cultured spheroids. (G) Western blot analysis of the expression of NICD in C45-4L-mCherry/C45-4S-EGFP chimeric spheroids treated with DMSO (0.1%) or DAPT (50 μM) for 7 days. (H and I) Spheroid-forming capacity (4H) and violin plots of the SSFG assay (4I) comparing the DMSO-treated EGFP-positive cells with the DAPT-treated C45-4L-mCherry/C45-4S-EGFP chimeric spheroids (Chimera), and the DMSO-treated mCherry-positive cells with the DAPT-treated chimeric cells. In Figure 4, the data of the spheroid-forming capacity were tested by one-way ANOVA, followed by Tukey’s test, and those of the SSFG assay were tested by the Mann–Whitney U test. ∗, p < 0.05.

To further reveal the transition, we conducted well-controlled experiments *in vitro*. Since the L-cells naturally generate the S-cells during spheroid formation while the S-cells did not generate the L-cells when isolated, we speculated that the transition mechanisms from the S- to the L-cells might depend on a cell-cell interaction, especially Notch signaling. Thus, we generated chimeric spheroids by aggregating the EGFP-labeled C45-4S cells (C45-4S-EGFP) and the mCherry-labeled C45-4L cells (C45-4L-mCherry) (Figures 5A and 5B) and subjecting them to the SSFG assay. The C45-4S-EGFP cells acquired the D-pattern phenotype within the chimeric spheroids, similar to the C45-4L-mCherry cells (Figures 5C and 5D). In contrast, the S-pattern was maintained in the C45-4S-EGFP cells when both the types of cells were co-cultured in different gel droplets within the same wells (Figures 5E and 5F), indicating that a close cell-cell interaction was necessary for this transition. To further investigate the molecular mechanisms of this cell-cell interaction, we examined Notch signaling, which was enriched in C45-4L spheroids (Figures 4H and 4I) and reportedly played an important role in determining the cell fate in the context of cell-cell contact.^30^^,^^31^ We inhibited Notch signaling with DAPT, a notch inhibitor, which was confirmed through Western blotting of NICD in the C45-4L-mCherry/C45-4S-EGFP chimeric spheroids (Figure 5G). The D-pattern of both parent C45 and C45-4L did not change through DAPT treatment (Figure S6A), indicating that Notch signaling does not affect the ability of L-cells to generate the D-pattern of spheroids or affect the growth of the spheroids. We, then, treated the C45-4L-EGFP and C45-4S-mCherry spheroids with DAPT and generated chimeric spheroids (Figure S6B). The treatment of DAPT did not affect the spheroid-forming capacity in either clone (Figure 5H); however, it inhibited the transition of the C45-4S-EGFP cells to the D-pattern within the chimeric spheroids (Figure 5I). Thus, the Notch signaling is involved in the transition from the S-cells to the L-cells. Meanwhile, delayed treatment of DAPT (Figure S6C) did not affect the transition (Figure S6D), indicating that Notch signaling was critical for the transition of S-cells to L-cells at early time point of the cell-cell contact with the L-cells.

To elucidate the role of Notch signaling on cell cycle reactivation in the S-cells-derived xenograft tumors, we evaluated the expression of HES1 in both mid- and end-point tumor xenografts as described above (Figure S7). Consistent with the PCNA staining results (Figure S5), the ratio of HES1-positive cells in the S-cells-derived xenograft tumors increased from approximately 2%–4% in the *in vitro* cultured S-pattern spheroids to an average of approximately 7%–10%, reaching the values of D-pattern spheroid-derived tumors (Figure S7). These results indicated that S-cells can also activate Notch signaling *in vivo*. Interestingly, the ratio of HES1-positive cells was higher at the midpoint than the endpoint, decreasing to approximately 5% at the endpoint of the S-cells-derived tumors, suggesting that the activation of Notch signaling is involved in the cell fate transition in S-cells *in vivo*.

### S-cells were the drug-resistant fraction

**Figure 6:**
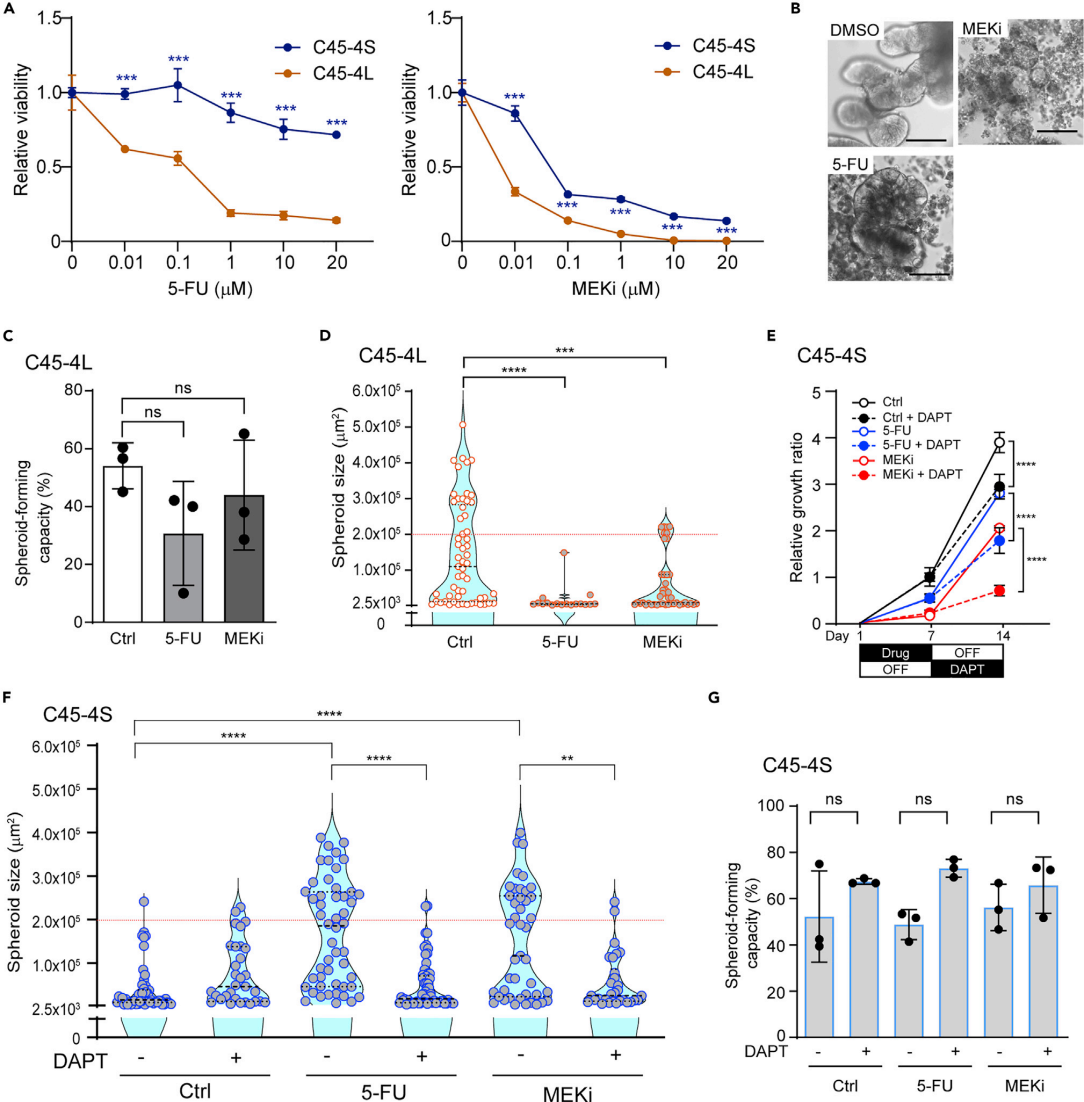
Transition from the drug resistant S-cells to the L-cells after drug exposure (A) Dose-dependent curves of the indicated C45 subclones treated with 5-FU (left) and MEKi (right) as evaluated by an ATP assay. The mean ± SD is shown, tested by two-way ANOVA, followed by Tukey’s test. (B) Representative phase-contrast images of the C45-4L spheroids (at day 7), treated with the indicated drugs. Scale bars: 100 μm. (C) Spheroid-forming capacity of C45-4L treated as indicated. (D) Violin plot of the SSFG assay for the C45-4L subclone, comparing the DMSO (0.1%)-treated with indicated drug-treated spheroids. (E) Time course of the growth of the non-treated (Ctrl) and the indicated drug-treated C45-4S spheroids. Relative size of the spheroids to the non-treated ones at day 1, 7, and 14 are shown. The period of the drug exposure and withdrawal is shown in black and white bars, respectively. The mean ± SD is shown, tested by two-way ANOVA, followed by Tukey’s test. (F) Violin plot of the SSFG assay for the C45-4S subclone, comparing the DMSO (0.1%)- and DAPT (50 μM) -treated with indicated drug-treated spheroids. (G) Spheroid-forming capacity of C45-4S treated as indicated. In Figure 5, the data of the spheroid-forming capacity were tested by one-way ANOVA, followed by Tukey’s test, and those of the SSFG assay were tested by the Mann–Whitney U test. Ns, not statistically significant. ∗∗, p < 0.01; ∗∗∗, p < 0.001; and ∗∗∗∗, p < 0.0001.

Next, to examine the drug sensitivity of the subpopulations with different growth pattern, we subjected them to the conventional sensitivity assay of organoids.^19^ We tested 5-FU (a drug that is currently used in clinical practice) and PD0325901 (a MEK inhibitor, and a candidate drug for CRC treatment.^32^ The S-pattern spheroids (C45-4S, C45-1, C45-2, C132-1, and KUC16-1) were significantly more resistant to both the drugs than the D-pattern spheroids (C45-4L, C45-3, C45-4, C132-2, and KUC16-2) (Figures 6A and S8), except for the C132 clones, which responded equally to PD0325901 (Figure S8). When the clones were exposed to higher doses of each drug, a few small and intact spheroids remained, even in the sensitive C45-4L spheroids (Figure 6B). We then performed the SSFG assays for the remaining small spheroids immediately after exposure to each drug. The MEKi treatment did not decrease the spheroid-forming capacities, and 5-FU made it approximately half, indicating that the remaining small spheroids retained substantial spheroid-forming capacity after drug exposure (Figure 6C). The SSFG assay revealed that the C45-4L cells had the S-pattern of spheroid growth (Figure 6D). After the removal of the drugs, the C45-4S clone grew in a similar manner to the non-treated spheroids (Figure 6E). When Notch signaling was inhibited by DAPT at the drug withdrawal time, the regrowth was suppressed (Figure 6E). The SSFG assay for the spheroids after drug withdrawal and regrowth revealed that the regrown spheroids of C45-4S switched to the D-pattern (Figure 6F). DAPT treatment diminished the switching to the D-pattern after drug withdrawal and regrowth in C45-4S. (Figure 6F). However, the spheroid-forming capacity did not change with the treatment of the drug or DAPT (Figure 6G). Taken together, the S-cells were the drug-resistant fraction, and the transition of the isolated S-cells to the L-cells occurred during the regrowth after the drug withdrawal depending on the Notch signaling.

### MSI1 was involved in the transition between the growth patterns of the cells

To further explore the genes that characterize the different phenotypes of spheroid growth, we selected 148 differentially expressed genes with relatively high intensity by a clustering and a heatmap analyses of the microarray data comparing the C45-clones and subclones (Figure S9A) (Table S5). Among the candidate genes, RNA-binding proteins, *MSI1* and *MEX3A*, were included. We focused on MSI1 since it has been reported that the Notch signaling regulates MSI1 expression in metastatic CRC cells (Pastò et al., 2014). In addition, MSI1 is reportedly a CSC marker in the normal intestine and the CRC,^33^^,^^34^ and is involved in the self-renewal of stem cells in both the normal intestine^35^ and CRC.^36^^,^^37^

**Figure 7:**
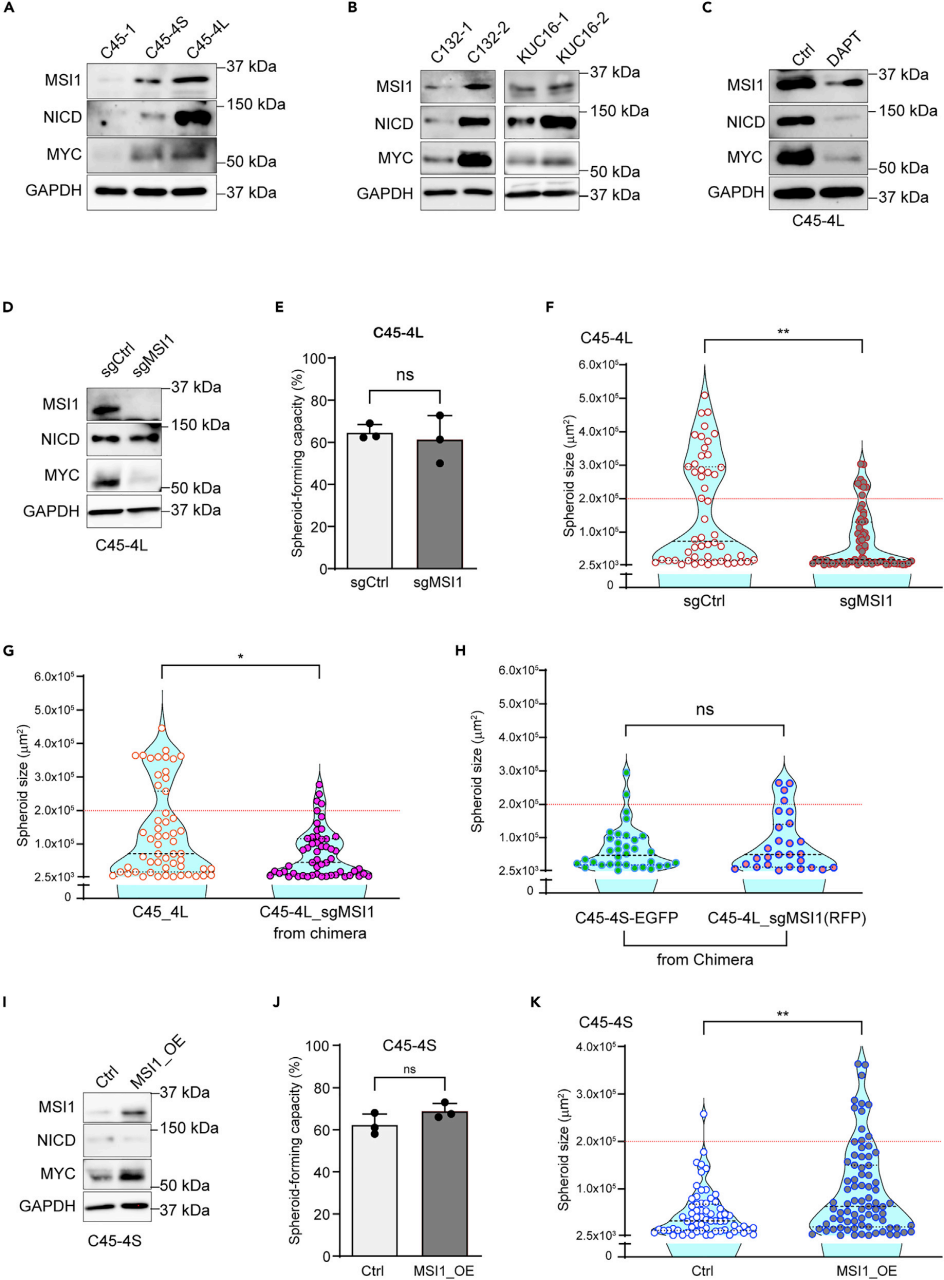
The RNA-binding protein MSI1 regulates the cell growth and plasticity of CRC spheroids (A–C) Western blot analyses of the MSI1, NICD, and MYC protein from indicated lines. (D) Western blot analyses of MSI1 and indicated protein levels in C45-4L spheroids infected with lentiviruses expressing Cas9 and the MSI1 sgRNA (sgMSI1) or non-targeting sgRNA (sgCtrl). (E and F) Spheroid-forming capacity (7E) and violin plot of the SSFG assay (7F) for the C45-4S subclones comparing the control and the *MSI1* knockout cells. (G) Violin plot of the SSFG assay for the C45-4L_sgMSI1-RFP cells derived from the chimeric spheroids mixed with C45-4L wild-type cells. (H) Violin plot of the SSFG assay for the C45-4S_EGFP and C45-4L_sgMSI1-RFP cells derived from the chimeric spheroids mixed with these cells. (I) Western blot analyses of MSI1 and other indicated proteins in C45-4S spheroids infected with a lentivirus that constitutively expresses MSI1 (MSI1_OE) or with the corresponding empty vector (Ctrl). (J and K) Spheroid-forming capacity (7J) and violin plot of the SSFG assay (7K) for the C45-4S subclones, comparing the control and the MSI1 overexpressing cells. In Figure 7, the data of the spheroid-forming capacity were tested by one-way ANOVA, followed by Tukey’s test, and those of the SSFG assay were tested by the Mann–Whitney U test. Ns, not statistically significant. ∗, p < 0.05. ∗∗, p < 0.01.

The expression levels of MSI1 were confirmed to be higher in the D-pattern spheroids (C45-4L) than in the S-pattern spheroids (C45-1 and C45-4S) in parallel with those of MYC and NICD (Figure 7A). This was also true in the subclones of other lines, such as C132 and the KUC16 (Figure 7B). Moreover, DAPT treatment suppressed the expression of not only NICD but also MSI1 and MYC (Figure 7C). To further investigate the functional role of MSI1 in the transition between the different growth patterns, we knocked out the MSI1 gene in the C45-4L spheroids by using the CRISPR/Cas9 system, generating the C45-4L_sgMSI1 spheroids (Figure 7D). No difference was observed regarding the spheroid-forming capacity (Figure 7E), while the ability of generating large spheroids was impaired (Figure 7F), thereby suggesting that MSI1 is not involved in the growth of the S-cells but in the transition of the growth pattern. We examined whether the cell-cell contact with the cells from D-pattern spheroids can rescue the impaired transition of the C45-4L_sgMSI1 cells to the D-pattern, as shown in Figure 5C. For this reason, we generated chimeric spheroids by mixing RFP-labeled C45-4L_sgMSI1 cells and wild-type C45-4L cells and subjected them to the SSFG assay. The C45-4L_sgMSI1 cells could not demonstrate their D-pattern phenotype, even in the chimeric spheroids with wild-type cells (Figure 7G), thereby suggesting that MSI1 is involved in the cell-cell contact-induced transition from the S- to the D-pattern of growth. We subsequently generated chimeric spheroids mixing EGFP-labeled C45-4S cells and RFP-labeled C45-4L_sgMSI1 cells, to check whether C45-4L_sgMSI1 cells retain the ability of parent C45-4L cells to switch the S-pattern spheroid (C45-4S) to the D-pattern in the chimera. Unlike the parent C45-4L, C45-4L_sgMSI1 cells failed to switch the S-pattern spheroids to the D-pattern (Figure 7H). We then overexpressed MSI1 in C45-4S and generated the C45-4S_MSI1OE spheroids (Figure 7I) and subjected to the SSFG assay. No difference was observed in their spheroid-forming capacity (Figure 7J), whereas growth of the C45-4S_MSI1OE showed the D-pattern (Figure 7K). Notably, not all the cells generated large spheroids. Similar results were obtained using another S-cell clone, KUC16-1 (Figures S9B–S9D). Taken together, MSI1 is not simply involved in the growth of the spheroids but functionally in the transition of the growth pattern.

The expression levels of MSI1 in the spheroids were accompanied with the increased protein levels of NICD and MYC in the clones with different growth patterns (Figures 7A and 7B), suggesting three molecules formed a network. Notch inhibition by DAPT in D-pattern spheroids (C45-4L) leads to a decrease of the MSI1 protein levels compared to the untreated control (Figure 7C), suggesting Notch signaling regulates the MSI1 expression. On the other hand, MSI1 reportedly blocks the translation of NUMB, which promotes the degradation of NICD,^38^ whereas neither the MSI1 knockout in D-pattern spheroids (C45-4L) nor the overexpression of MSI1 in S-pattern spheroids (C45-4S) affected the NICD protein levels (Figures 7D and 7I), suggesting MSI1 does not regulate Notch signaling in this experimental context. For the MYC expression, the *MSI1* knockout in C45-4L resulted in a decrease in the MYC protein levels (Figure 7D), meanwhile the MSI1 overexpression in the C45-4S spheroids resulted in an increase of the MYC protein levels (Figure 7I). Taken together, MSI may be located downstream to Notch and upstream to MYC.

## Discussion

Our experiments revealed that the patient-derived CRC organoids consist of phenotypically heterogeneous and interchangeable spheroid-forming cells with different cell fate of growth and drug sensitivity. At the second-round spheroid-forming assay, the cells in a small spheroid gave rise to only S-pattern, whereas the cells in a large spheroid gave rise to D-pattern. The transition from the S- to the D-pattern is molecularly regulated, and Notch signaling and MSI1 play a significant role in it. Our data provide new insights into the molecular mechanisms regulating cancer cell heterogeneity and plasticity.

In this study, we applied a method that allowed us to precisely track the capacity of not only spheroid formation but also the growth of each spheroid-forming cell in CRC organoids with a single-cell resolution: the SSFG assay. The spheroid-forming capacity was varied, but it was generally high in this study, ranging from 19% to 59% (Table S2). Interestingly, the spheroid-forming capacity was similar among the studied clones and subclones, a finding that supports the idea that the variation in spheroid growth is an event that occurs within cell populations that demonstrate spheroid-forming ability.

Herein, we demonstrated that the CRC cells consist of two distinct but interchangeable subpopulations. The juxtacrine interaction (or cell-cell interaction) with the D-pattern cells as well as the tumor microenvironment was found to be critical in order for the S-pattern cells to transit to the D-pattern phenotype. Our results have also suggested the existence of a gate^39^^,^^40^ that regulates the transition from the S- to the D-pattern in CRC cells through a nongenetic process. In fact, Notch signaling and MSI1 seem to be the regulators of this transition.

Since all of the clones studied here were able to form spheroids at a high frequency and be tumorigenic, the existence of the subclones with different type of growth patterns and their interchangeable nature might be relevant to CSC, although the growth-permissive culturing conditions^18^ in the SSFG assay in this study were different from those of earlier studies on CSCs from CRC.^41^^,^^42^ Indeed, recent studies have revealed that stem-like properties of cancer cells can dynamically fluctuate.^5^^,^^10^^,^^15^^,^^40^ In addition, many of the reported CSC markers in CRC were in the list of the differentially expressed genes between the clones with different type of growth patterns. Notably, the increase or decrease depended on each marker.

An overexpression of MSI1 has been reported in different tumor types,^43^ including CRCs,^36^ and MSI1 has been described as a CSC marker in CRC.^33^^,^^34^ We, herein, demonstrated that the expression levels of MSI1 were lower in the S-pattern spheroids, and could functionally modulate the transition of the growth status in the studied spheroids (Figure 7). Notably, the spheroid-forming capacity was affected by neither the gene knockout nor the overexpression of MSI1, thus indicating that MSI1 is not essential for the cells to be stem-like. In previous reports, the knockdown of *MSI1* in CRC cells suppressed their capacity of spheroid formation and their tumorigenicity.^36^^,^^37^^,^^44^ However, in these cases, the number of spheroids could have been underestimated because of the increased slow-growing populations, and the fact that the MSI1-downregulated cells in these studies did not actually lose their ability to form tumors, but they rather exhibited growth retardation. The molecular mechanisms by which the phenotypically different subpopulations are generated in the D-pattern cells remain to be elucidated. One possible mechanism is that of asymmetric division,^30^^,^^45^^,^^46^ as MSI1 was originally reported to play a role in asymmetric division.^47^

The majority of current cancer therapies have been designed and developed against fast-growing cancer cells, even when CSCs are targeted.^9^ This is despite the fact that resistance to these anticancer therapies has been repeatedly linked to the presence of quiescent or slow-growing CSCs.^8^^,^^48^ Given that CSCs fluctuate between different growth states, therapeutic anticancer strategies targeting CSCs should be seriously revisited.^5^^,^^10^^,^^13^^,^^21^^,^^22^^,^^23^^,^^40^ In this study, we revealed that the slow-growing cells play an important role in drug resistance, and that the two identified subpopulations are interchangeable.

The concept of the “drug-tolerant persister” (DTP) has recently emerged as an important driver of therapy failure and tumor relapse. A DTP is a cancer cell that is characterized as being quiescent or slow-cycling.^23^ Cancer cells may enter a nongenetic and reversible DTP state to evade cellular death from conventional chemotherapies or molecular-targeted therapies.^49^^,^^50^^,^^51^ The S-cell showed phenotypically DTP-like status in CRC organoids when treated with chemotherapeutic drugs. The isolated S-cells could be a novel platform for investigating DTPs and developing the DTP targeting treatment. Study of the transition to other fates, especially to the non-growing spheroids in future would provide more attractive targets for cancer therapy.

### Limitation of the study

While our spheroid formation and growth assay with the single-cell resolution revealed the existence of two distinct but interchangeable subpopulations in CRC tumors and organoids, there are some limitations in this study. First, the assays *in vitro* generally run the risk of making the cells adapted to the culture conditions. Therefore, characteristics of these subpopulations in both patients' tumors and patient-derived tumor xenografts remain to be elucidated. In addition, CRC organoids showed S-pattern of growth when treated with chemotherapeutic drugs, while it should be further clarified how similar or different these spheroids are compared with the DTP cells *in vivo*. Investigating these issues will contribute to the clinical application of the findings in this study.

## STAR★Methods

### Key resources table

**Table:** 

REAGENT or RESOURCE	SOURCE	IDENTIFIER
**Antibodies**		

Akt (pan) (40D4)	Cell Signaling Technologies	Cat #: 2920, RRID: AB_1147620
Anti-mouse IgG	Cell Signaling Technologies	Cat #: 7076, RRID: AB_330924
Anti-rabbit IgG	Cell Signaling Technologies	Cat #: 7074, RRID: AB_2099233
c-Myc (D84C12)	Cell Signaling Technologies	Cat #: 5605, RRID: AB_1903938
Cleaved Notch1 (Val1744) (D3B8)	Cell Signaling Technologies	Cat #: 4147, RRID: AB_2153348
GAPDH (14C10)	Cell Signaling Technologies	Cat #: 2118, RRID: AB_561053
MEK1/2	Cell Signaling Technologies	Cat #: 9122, RRID: AB_823567
Msi1 antibody [EP1302]	Abcam	Cat #: ab52865, RRID: AB_881168
p44/42 MAPK (Erk1/2) (3A7)	Cell Signaling Technologies	Cat #: 9107, RRID: AB_10695739
Phospho-Akt (Ser473)	Cell Signaling Technologies	Cat #: 9271, RRID: AB_329825
Phospho-MEK1/2 (Ser217/221)	Cell Signaling Technologies	Cat #: 9121, RRID: AB_331648
Phospho-p44/42 MAPK (Erk1/2) (Thr202/Tyr204) (D13.14.4E)	Cell Signaling Technologies	Cat #: 4370, RRID: AB_2315112
Phospho-S6 Ribosomal Protein (Ser235/236)	Cell Signaling Technologies	Cat #: 2211, RRID: AB_331679
Ribosomal S6 Protein	Cell Signaling Technologies	Cat #: 2212, N/A
PCNA (PC10)	Cell Signaling Technologies	Cat #: 2586, RRID: AB_2160343
Alexa Fluor™ 555 Goat anti-Mouse IgG (H+L)	Thermo Fisher Scientific	Cat #: A-21422, RRID: AB_2535844

**Chemicals, peptides, and recombinant proteins**		

2-Mercaptoethanol	Fujifilm	137-06862
5-FU	Kyowa Kirin	N/A
Blasticidine S hydrochloride	Millipore Sigma	15205
D-MEM/Ham’s F-12 with L-Glutamine, Phenol Red, HEPES and Sodium Pyruvate	Fujifilm	042-30555
DAPT, gamma-Secretase inhibitor	Abcam	ab120633
Dimethyl sulfoxide (DMSO) ReagentPlus®, ≥99.5%	Millipore Sigma	D5879
DNase I	Roche	11284932001
HBSS(+) without Phenol Red	Fujifilm	084-08965
Liberase™ DH Research Grade, high Dispase concentration	Roche	5401089001
Matrigel® Basement Membrane Matrix	Corning	354234
Matrigel® Growth Factor Reduced (GFR) Basement Membrane Matrix	Corning	354230
Mirdametinib (PD0325901)	Selleckchem	S1036
Penicillin-Streptomycin (10,000 U/mL)	Thermo Fisher Scientific	15140122
StemCell Keep	BioVerde	BVD-VPL-A1-20
StemPro™ hESC SFM	Thermo Fisher Scientific	A1000701
Trypsin-EDTA (0.25%)	Thermo Fisher Scientific	25200072
Y-27632	Selleckchem	S1049
ProLong™ Gold Antifade Mountant with DAPI	Thermo Fisher Scientific	P36931
Propidium Iodide	Thermo Fisher Scientific	P3566
Hoechst 33342	Thermo Fisher Scientific	H3570
RNAscope® Probe Hs-HES1	Advanced Cell Diagnostics	311191
SCALEVIEW-S4	Fujifilm	194-18561
Bovine Serum Albumin	Sigma-Aldrich	A3059

**Critical commercial assays**		

CellTiter-Glo® Luminescent Cell Viability Assay	Promega	G7570
Fast SYBR™ Green Master Mix	Thermo Fisher Scientific	4385617
Gateway™ LR Clonase™ II Enzyme mix	Thermo Fisher Scientific	11791020
PrimeScript™ 1st strand cDNA Synthesis	Takara	6210A
PrimeSTAR® Max DNA Polymerase	Takara	R045A
RNase-Free DNase Set	QIAGEN	79254
RNeasy Mini Kit	QIAGEN	74106
SuperScript™ III Reverse Transcriptase	Thermo Fisher Scientific	18080085
X-tremeGENE™ HP DNA	Roche	6366236001
RNAscope 2.5 HD Reagent Kit-BROWN	Advanced Cell Diagnostics	322300

**Deposited data**		

Gene expression microarray	This study	GEO: GSE185012

**Experimental models: Cell lines**		

Human: colorectal organoids (CTOSs): see Tables S1–S3	This study	N/A
Human: single-cell originated spheroids derived from CRC CTOSs	This study	N/A

**Experimental models: Organisms/strains**		

NOD/Scid mice	CLEA Japan	NOD/ShiJic-scidJcl

**Oligonucleotides**		

Primers and single-guide RNA oligos: see Table S6	This study	N/A

**Recombinant DNA**		

7TFC-mCherry	Addgene	#24307
pDONR221_EGFP	Addgene	#25899
pENTR4 no ccDB	Addgene	#17424
pL-CRISPR.SFFV.tRFP	Addgene	#57826
pLX304	Addgene	#25890
pMD2.G	Addgene	#12259
psPAX2	Addgene	#12260

**Software and algorithms**		

GPP sgRNA Designer	Broad Institute	https://portals.broadinstitute.org/gpp/public/analysis-tools/sgrna-design
GraphPad Prism 9	GraphPad Software Inc.	https://www.graphpad.com/
GSEA	Broad Institute	https://www.gsea-msigdb.org/gsea/index.jsp
ImageJ (Fiji)	https://doi.org/10.1038/nmeth.2019	https://imagej.net/software/fiji
LAS X Life Science	Leica Microsystems	https://www.leica-microsystems.com/products/microscope-software/p/leica-las-x-ls/downloads/
LuminaVision	Mitani	https://www.mitani-visual.jp/download/catalogs/
CellSens	Olympus	https://www.olympus-lifescience.com/en/software/cellsens/
R	The R Project for Statistical Computing	https://www.r-project.org/
RStudio	RStudio	https://www.rstudio.com/

**Other**		

24-well plate, flat bottom, non-treated	Iwaki	1820-024
6-well plate, flat bottom, non-treated	Iwaki	1810-006
96F UNTREATED STRAIGHT W/LID	Thermo Fisher Scientific	260860
Cellstar® 96U-well plate	Greiner Bio-One	650185
ClipTip™ 384 125	Thermo Fisher Scientific	94410153
E1-ClipTip™ Equalizer	Thermo Fisher Scientific	4672060BT
Falcon® 5 mL Round Bottom Polystyrene Test Tube, with Cell Strainer Snap Cap	Corning	352235
Falcon® Cell Strainers	Corning	352340; 352350; 352360
PrimeSurface® 384U	Sumitomo Bakelite	MS-9384U
Syringe 27Gx1/2 (1 mL)	Terumo	SS-10M2713

### Resource availability

#### Lead contact

Further information and requests for resources and reagents should be directed to and will be fulfilled by the lead contact, Masahiro Inoue (masa_inoue@kuhp.kyoto-u.ac.jp).

#### Materials availability

• This study did not generate new unique reagents.
• There are restrictions to the availability of CRC CTOS lines through Material Transfer Agreement requirements at Kyoto University specific to the CRC CTOS and the subclones.

### Experimental model and subject details

#### Preparation and culture of tumor organoids

The study was approved by the Institutional Ethics Committees at Osaka International Cancer Institute (1803125402) and Kyoto University (R1575, R1671). Fresh surgical samples from CRC patients were obtained with the patients’ written informed consent. CRC organoids were prepared from patient tumor samples or xenografts (18,19). Briefly, tumors were mechanically minced and incubated for 30-45 min in DMEM/Ham’s F12 medium (Fujifilm, 042-30555) containing Liberase DH (Roche, 5401089001) at a final concentration of 0.26 U/mL at 37°C with continuous stirring. DNase I (Roche, 11284932001) was added at 10 μg/mL, followed by an additional 15-min incubation. The digested solution was serially strained using mesh filters (Falcon Cell Strainers). Tumor fragments of the 250-500, 100-250, and 40-100 μm fractions were recovered and cultured for 24h in the CTOS organoid medium: DMEM/F-12 with 1x GlutaMAX, 1x StemPro hESC, 1.8% BSA (StemPro hESC SFM, Thermo Fisher Scientific, A1000701), and 100 U/mL P-S (Thermo Fisher Scientific, 15140122) in non-treated plates (IWAKI, 1810-006). The next day, organoids were washed with HBSS (Fujifilm, 084-08965) to remove cellular debris and cultured either in suspension or embedded in Matrigel GFR (Corning, 354230) in the CTOS organoid medium. The organoids prepared from freshly harvested primary CRC tumors (KUCs) were cultured and expanded for 14-21 days in the CTOS organoid medium and subjected to the experiments. For *in vitro* passages, organoids were dissociated once a week by the syringe disruption method.^52^ Briefly, organoids or spheroids were disrupted into smaller fragments by passing them through a 1 mL syringe with a 27 G needle (Terumo, SS-10M2713) at a high flow rate (∼30 mL/min). Organoids were spontaneously re-formed from these fragments. All experiments were performed at least one day after passaging to avoid the influence of the disruption and remodeling of the spheroids. Within one month of culture, organoids were freeze-stocked with StemCell Keep (BioVerde, BVD-VPL-AI-20). ‘CRC organoid lines’ were defined by the following criteria: i) growing continuously in culture, ii) generating xenograft tumor (at least 2 passages *in vivo*), and iii) being sufficiently freeze stocked in order to reproduce the experiments. Table S1 presents clinical details regarding the 14 organoid lines in the CRC panel.

#### Xenotransplantation of spheroids

The animal studies were approved by the Institutional Animal Care and Use Committee of Osaka International Cancer Institute (16062411, 17052610, 18060708) and Kyoto University (18564). They were performed in compliance with the institutional guidelines. To generate xenograft tumors of CRC organoid lines, 2 × 10^3^ organoids were suspended in a 1:1 mixture of medium and Matrigel (Corning, 354234) and subcutaneously injected into the flank of NOD/Scid mice (4-5 weeks old) (CLEA Japan). When tumor volume reached ∼1 cm^3^, mice were sacrificed. For the *in vivo* tumor growth assay, 1 × 10^3^ organoids with similar size (diameter: 40-70 μm) and shape were used for inoculation. Tumor growth was monitored every 2-3 days. Tumor volume was calculated using the following formula: 0.5 x width^2^ x length. For the *in situ* hybridization and the tissue immunofluorescence staining analyses, the tumor xenografts were collected in two different time points: a (i) midpoint, when the tumor volume was detectable for the first time; and a (ii) endpoint, when the tumor volume reached ∼200 mm^3^.

#### Single-cell-derived sphere-forming and growth (SSFG) assay

A hundred to a thousand organoids were collected and dissociated into single cells by treating with 0.25% Trypsin-EDTA (Thermo Fisher Scientific, 25200072) and DNase I (10 μg/mL) for 10 min at 37°C and 1 min at room temperature, respectively. Then, the cell suspension was gently pipetted a hundred times to promote cell dissociation and filtered through a 35 μm cell strainer (Corning, 352235) to remove cell clusters. The dissociated single cells were diluted in the SSFG medium: CTOS organoid medium containing 2% Matrigel GFR and 10 μM of a ROCK inhibitor, Y-27632 (Selleckchem, S1049), and seeded in a non-treated 384-well plate (Sumitomo Bakelite, MS-9384U) with a ratio of 1 cell per well (50 μL/well) using an E1-ClipTip electronic pipette (Thermo Fisher Scientific, 4672060BT). Within 2 h after cell seeding (day 0), the presence of one cell per well was confirmed by image acquisition using the LEICA DMI4000B microscope (Leica Microsystems) equipped with Lumina Vision (Mitani Corporation). Single cells with an area greater than 300 μm^2^ (small cluster) on day 0 and wells containing multiple cells were excluded from the subsequent analyses. The growth of single cells into spheroids was monitored by image acquisition. Fresh SSFG medium without Y-27632 (30 μL/well) was added on day 7. The ability to form spheroids as well as the growth were evaluated on day 13 for C45 clones and on day 20 for other lines unless otherwise noticed, by measuring the area of each single-cell-derived spheroid using the acquired pictures and ImageJ Fiji software (https://imagej.net/software/fiji). Spheroid-forming capacity was calculated and expressed as the percentage of single cells able to grow and form spheroids. Mean ± SD from at least three independent experiments is shown. For the experiments with the organoids (C45-1, C45-4S, and C45-4L) derived from freshly harvested xenograft tumors, we prepared organoids from the xenografts and subjected them to the SSFG assay within 6 h of preparation. For the experiments with chimeric spheroids, the single cell-derived spheroids with different fluorescence were assessed by image acquisition using the LEICA DMi8 microscope and LAS X software (Leica Microsystems). For the DAPT treatment experiments, tumor organoids (C45 and C45-4L) were treated with 50 μM of DAPT (Abcam, ab120633) or 0.1% of DMSO (Millipore Sigma, D5879) as control, cultured for 7 days, and subjected to the SSFG assay.

### Method details

#### Isolation and culture of the slow- and dual-growing spheroids

At the first round of the SSFG assay, the selected single-cell-derived slow- and fast-growing spheroids were picked up and individually cultured and expanded *in vitro* in the CTOS organoid medium. A subsequent round of SSFG assay was performed for each selected tumor spheroid clone to evaluate its growing pattern: slow- or dual-growing phenotype. For the additional rounds of the SSFG assay, the assay was sequentially performed for each clone and indicated as round one (x1), two (x2), and three (x3). Between the rounds, a pool of spheroids with similar size was collected and subjected to the next round of the SSFG assay.

#### Chimeric spheroids and co-culture system

To generate chimeric spheroids, fluorescent-labeled spheroids were dissociated into single cells and mixed at a 2:1 ratio (1 × 10^4^ cells of EGFP-labeled C45-4S: 5 × 10^3^ cells of mCherry-labeled C45-4L; or 1 × 10^4^ cells of tRFP-labeled sgRNA MSI1-C45-4L: 5 × 10^3^ cells of wild-type C45-4L; or 1 × 10^4^ cells of EGFP-labeled C45-4S: 5 × 10^3^ cells of tRFP-labeled sgRNA MSI1-C45-4L). The cell mixture was suspended in the SSFG medium and dispensed into the round-bottom non-treated 96-well plate (Greiner bio-one, 650185). The plate was centrifuged at 400x g for 3 min to facilitate aggregation and incubated for 7 days. The chimeric spheroids were collected and subjected to the SSFG assay. For the DAPT treatment experiments, the fluorescent-labeled spheroids were pretreated overnight with 50 μM of DAPT or 0.1% of DMSO as control. Then, the chimeric spheroids were generated in the presence of DAPT (50 μM) or DMSO (0.1%), cultured for 7 days, and subjected to the SSFG assay. For the delayed treatment with DAPT, the chimeric spheroids were treated with 50 μM of DAPT or DMSO (0.1%) at day 2 of culture, and then cultured and subjected to the SSFG assay as described above. For the co-culture system without physical cell-cell interaction, 1 × 10^3^ cells for each clone were separately embedded in 7 μL of Matrigel GFR, solidified as a droplet in a non-treated 24-well (IWAKI, 1820-024), overlaid with CTOS organoid medium containing 10 μM Y-27632, and cultured for 7 days. The EGFP-labeled-C45-4S spheroids were collected and subjected to the SSFG assay. For control experiments, the same number of EGFP-labeled C45-4S cells were cultured alone in the same way.

#### Organoid drug sensitivity assay

The organoid drug sensitivity assay,^19^ spheroids with similar size and shape (diameter: 40-100 μm) were collected and seeded in non-treated 24-well plates at a density of 1 × 10^2^ per well. Spheroids were cultured for one week in the CTOS organoid medium containing the indicated dose of the drugs or 0.1% of DMSO as control (n = 3 wells for each condition). Pictures of the entire well were captured on day 0 and day 7. The viabilities of the spheroids were evaluated using the CellTiter-Glo assay (Promega, G7570). ATP content was measured at day 7 and adjusted to the control group. The replicas of the spheroids at day 7 were subjected to the SSFG assay. The 5-FU (Kyowa Kirin Co., Ltd) compound was provided by the Department of Pharmacy, Osaka International Cancer Institute. The PD0325901 MEK1/2 inhibitor (Selleckchem, S1036) was used in this study.

#### Spheroid-forming cells treatment and regrowth assay

Tumor spheroids were dissociated into single cells as described before. Subsequently, 5 × 10^2^ cells were embedded in 7 μL of Matrigel GFR and solidified as a droplet in non-treated 96-well plates (Thermo Fisher Scientific, 260860) with a ratio of one drop per well. Upon solidification, dispersed single cells were cultured for 7 days in 100 μL of CTOS organoid medium containing 10 μM of Y-27632 and 1 μM of the the indicated drugs (5-FU, PD0325901) or DMSO (0.1%) as control (n = 3 ± 4 wells for each condition). Reconstituted spheroid viability was evaluated using the CellTiter-Glo assay. ATP content was measured at day 1, day 7, and adjusted to the control group. For the regrowth assay, the medium containing the indicated compound was removed at day 7 of culture. Then, each well was washed twice with HBSS and fresh CTOS organoid medium was added (100 μL). Spheroids were cultured for an additional week (day 14). The spheroid regrowth was assessed by measuring ATP content (CellTiter-Glo assay) at day 14 of culture and adjusted to the control group. Pictures of the entire well were captured on day 1, day 7 and day 14. For the DAPT treatment at the timing of the drug withdrawal, spheroids were treated with 50 μM of DAPT or DMSO (0.1%) at day 7, and the spheroid regrowth was evaluated at day 14 of culture as described above. The replicas of the spheroids at day 14 were subjected to the SSFG assay.

#### Cell death assay

To detect the cell death, tumor spheroids were cultured in non-treated 24-well plates, at a density of 0.5 × 10^2^ per well, and incubated with Propidium Iodide (PI) (P3566, Thermo Fisher Scientific), that is not permeant to live cells, and Hoechst 33342 (P3570, Thermo Fisher Scientific) at 37°C for 1 h. Next, fluorescence images of the spheroids were assessed using the LEICA DMi8 microscope and LAS X software (Leica Microsystems). The area of PI-positive cells was evaluated using ImageJ (Fiji) software (https://imagej.net/software/fiji), normalized per total nuclei area, and shown as a percentage. A total of five fields per well were examined for each clone.

#### Vector construction and gene transfer

The following lentivirus-expressing vectors were used: 7TFC-mCherry (Addgene #24307, a gift from Roel Nusse); pLX304 (Addgene #25890, a gift from David Root); and pL-CRISPR.SFFV.tRFP (Addgene #57826, a gift from Benjamin Ebert). For generating the pLX304-EGFP vector, the EGFP sequence was transferred from the pDONR221_EGFP plasmid (Addgene #25899, a gift from David Root) into the pLX304 plasmid by the Gateway cloning system (Thermo Fisher Scientific, 11791020). For MSI1 overexpression, human MSI1 cDNA was amplified with the primers of MSI1_BamHI-F and MSI1_stopdead_XbaI-R (Table S6) using the PrimeScriptTM first strand cDNA Synthesis (Takara, 6210A) and PrimeSTAR^Ⓡ^ Max DNA Polymerase kits according to manufacturer instructions (Takara, R045A). The PCR product was cloned into the pENTR4 no ccDB plasmid (Addgene #17424, a gift from Eric Campeau & Paul Kaufman) and transferred into the pLX304 plasmid by the Gateway cloning system. The lentiviral vectors (Roche, 6366236001; Addgene #12259 and #12260, both gifts from Didier Trono) using the X-tremeGENE HP DNA kit (Roche, 6366236001) according to the manufacturer’s protocol.^53^ For the pLX304-GFP and -MSI1, blasticidin (2 μg/mL) (Millipore Sigma, 15205) selection was performed. For the pLX304-EGFP and 7TFC-mCherry, the green (EGFP)- and red (mCherry)-positive cells from the reconstituted spheroids, respectively, were sorted and expanded. For *MSI1* knockout, single-guide RNA (sgRNA) or a non-targeting (sgCtrl) sgRNA was cloned into a pL-CRISPR.SFFV.tRFP expression vector (Addgene #57826, a gift from Benjamin Ebert) as described by Heckl et al.^54^ The sgRNA sequences were designed using the Broad Institute sgRNA designer tool (http://portals.broadinstitute.org/gpp/public/analysis-tools/sgrna-design). The sequences of the sgRNA oligos are shown in Table S6. Single cells derived from C45-4L spheroids were transduced with the lentiviral Cas9-expression vector (pL-CRISPR.SFFV.tRFP) containing the MSI1 sgRNA or the non-targeting sgRNA as control. The red (tRFP)-positive cells from the reconstituted spheroids were sorted and expanded.

#### RNA isolation and semi-quantitative real-time PCR

Spheroids were cultured for 7 days from single cells embedded in Matrigel GFR (5 × 10^2^ cells/7 μL of Matrigel GFR) in CTOS organoid medium containing 10 μM of Y-27632. Total RNA was extracted with the RNeasy mini kit (Qiagen, 74106) plus on-column DNAse I digestion (Qiagen, 79254). Semi-quantitative real-time PCR was carried out using Fast SYBR Green Master Mix and SuperScript III Reverse Transcriptase kits according to the manufacturer’s protocol (Thermo Fisher Scientific, 18080085 and 4385617, respectively).^55^ Data are presented as the mean ± SD of three replicates. The primer sequences are presented in Table S6.

#### Microarray and GSEA analysis

Spheroids were cultured for 7 days from single cells embedded in Matrigel GFR (5 × 10^2^ cells/7 μL of Matrigel GFR) in CTOS organoid medium containing 10 μM of Y-27632. Total RNA was extracted as described before. Microarray analysis, from three biological replicates, was performed with the GeneChip Human Gene 2.0 ST Array. Signals were quantified and normalized with the RMA algorithm. For gene set enrichment analysis (GSEA), software was downloaded from the Gene Set Enrichment Analysis website (http://www.broad.mit.edu/gsea/downloads.jsp). GSEA was performed using the c-Myc target^27^ and the Notch signaling pathway (PID_NOTCH_PATHWAY^29^) gene sets to identify enriched/depleted signatures. Gene sets with an FDR< 0.25 and a nominal p-value of <0.05 were considered significant. Volcano plots were generated with the GraphPad Prism version 9 (GraphPad Software) using the transcriptome data and setting the threshold for the log_2_(Fold Change) as ± 0.59 (Fold change <0.67 = −0.59; Fold change >1.5 = +0.59) and − Log_10_(p value) as 1.3 (>1.3 = p < 0.05). To generate a heatmap, gene expression data of day 8 samples were analyzed. After omitting the probes without gene name, the probes with average expression level at top 40% were extracted. Then, FDR was calculated for 6 slow samples vs 3 fast samples. Probes with FDR<0.1 were extracted to generate heatmap (heatmap3: R version 3.6.3 (http://www.R-project.org)).

#### Western blot analysis

Spheroids in suspension or single cell-derived spheroids embedded in Matrigel GFR (5 × 10^2^ cells in 7 μL of Matrigel GFR) were cultured for 7 days in the CTOS organoid medium. Western blotting analyses were performed^55^ using the following antibodies; The total AKT (#2920), pAKT-S473 (#9271), MEK1/2 (#9122), pMEK1/2-S217/221 (#9121), ERK1/2 (#9107), pERK1/2-T202/Y204 (#4370), GAPDH (#2118), S6 (#2212), p-S6 (#2211), c-Myc (#5605), and NICD (#4147) antibodies were obtained from Cell Signaling Technology. MSI1 (ab52865) antibody was obtained from Abcam. Secondary anti-rabbit (#7074) and -mouse (#7076) antibodies were obtained from Cell Signaling Technology. All antibodies were used at the producer’s suggested concentrations. For the DAPT treatment experiments, C45-4L spheroids were treated with 50 μM of DAPT (Abcam, ab120633) or 0.1% of DMSO (Millipore Sigma, D5879) as control, cultured overnight, and subjected to Western blotting analysis.

#### Immunofluorescence

For the whole-mount immunofluorescence staining, the tumor spheroids were collected and fixed with acetone/methanol (1 : 1) at 4°C for 45 min, and permeabilized with PBS containing 1% Triton X-100 at room temperature for 30 min. After blocking with PBS containing 2.5% BSA (A3059, Sigma-Aldrich) and 0.2% Triton X-100 (blocking buffer) for 30 min, spheroids were incubated with PCNA antibody (#2586, Cell Signaling Technologies) in blocking buffer at 4°C for 48 h. Next, the primary antibody was removed, and samples were washed three times with PBS containing 0.5% BSA and 0.2% Triton X-100 (washing buffer). The secondary anti-mouse antibody conjugated with Alexa 555 (A-21422, Thermo Fisher Scientific) and Hoechst 33342 (P3570, Thermo Fisher Scientific) were added to the spheroids and incubated at 4°C for 48 h. Finally, samples were washed three times with the washing buffer and the spheroids were mounted with SCALEVIEW-S4 (194-18561, Fujifilm). Fluorescence images were obtained using a Leica TCS SPE confocal microscope (Leica Microsystems). The number of PCNA-positive cells was evaluated using ImageJ (Fiji) software (https://imagej.net/software/fiji), normalized per total nuclei number, and shown as a percentage. Three to five spheroids, ten Z-stacks with 10 μm distance of each spheroid, were examined. The tissue immunofluorescence staining of tumor xenografts with PCNA and DAPI (P36931, Thermo Fisher Scientific) were performed as described before.^18^ Images were acquired using the Olympus BX50 Fluorescence Microscope and CellSens standard imaging software (Olympus). The number of PCNA-positive cells was evaluated as described above, and a total of ten fields for each tumor xenograft were examined.

#### *In situ* hybridization

*In situ* hybridization was performed in both tumor spheroids and tumor xenografts using the RNAscope 2.5 HD Reagent Kit—BROWN (322300) and the RNAscope Probe - Hs-HES1 (311191) (Advanced Cell Diagnostics) according to the manufacturer’s protocol. The area of HES1-positive cells was calculated using ImageJ (Fiji) software (https://imagej.net/software/fiji) according to the manufacturer’s protocol (https://acdbio.com/system/files_force/gated/TS_46_003_Tech_Note_ImageJ_04112019.pdf?download=1) and shown as a percentage. A total of ten fields for each tumor xenograft and five tumor spheroids for each C45 clone were examined.

### Quantification and statistical analysis

Statistical analyses were carried out with GraphPad Prism 9 (GraphPad Software). Significance was tested with the unpaired Student’s *t* test for single comparisons, and with one-way or two-way ANOVA analysis followed by Tukey’s or Bonferroni’s test for multiple comparisons as indicated. For the analyses of SSFG assay results that did not show a normal distribution, a non-parametric test, the Mann-Whitney test, was used. p-values <0.05 were considered significant. Violin plots showing the area of growing spheroids and frequency distribution analysis of their size (indicated as a percentage) were performed using the GraphPad Prism version 9. Representative data of three independent experiments are shown for the violin plots. Bimodal distribution was tested by Silverman’s bootstrap test with the null hypothesis that the kernel density has one mode, ∗p < 0.1, using VISUAL-SILVERMAN.^56^ Ns, not statistically significant; ∗, p < 0.05; ∗∗, p < 0.01; ∗∗∗, p < 0.001; and ∗∗∗∗, p < 0.0001.

## Data Availability

• The gene expression microarray data have been deposited at GEO and are publicly available as of the date of publication. Accession number is listed in the key resources table. • Any additional information required to reanalyze the data reported in this paper is available from the lead contact upon request. • This paper does not report original code.

## Supplemental information

Document S1. Figures S1–S9, Tables S1–S3 and S6

Table S4. List of differentially-expressed genes between C45-4L and C45-1, C45-4L and C45-4S, C45-4S and C45-1, related to Figure 4 Upregulated genes (fold change <0.67, p < 0.05) and downregulated genes (fold change >1.5, P < 0.05) are shown in each case.

Table S5. List of differentially-expressed genes in C45-4L commonly compared to C45–1 and C45-4S, related to Figure 4
