# Supplementary material for: Distinct but interchangeable subpopulations of colorectal cancer cells with different growth fates and drug sensitivity
Source: iScience. 2023 Jan 13;26(2):105962. doi: 10.1016/j.isci.2023.105962 (PMC9883198; doi:10.1016/j.isci.2023.105962)
Supplement: Document S1. Figures S1–S9, Tables S1–S3 and S6 [file mmc1.pdf]

iScience, Volume *26*

Supplemental information

Distinct but interchangeable subpopulations of colorectal cancer cells with different growth fates and drug sensitivity

Roberto Coppo, Jumpei Kondo, Keita Iida, Mariko Okada, Kunishige Onuma, Yoshihisa Tanaka, Mayumi Kamada, Masayuki Ohue, Kenji Kawada, Kazutaka Obama, and Masahiro Inoue

Figure S1

S1A

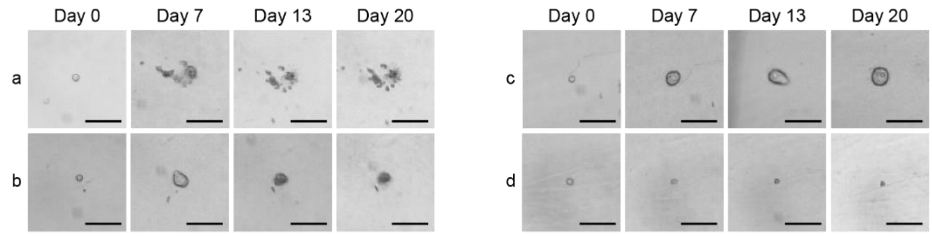

S1B

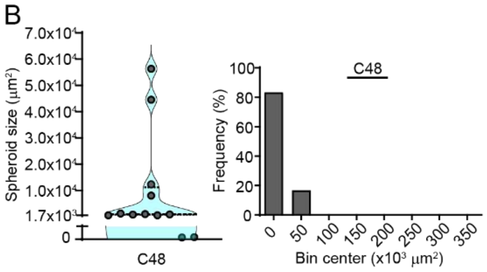

S1C

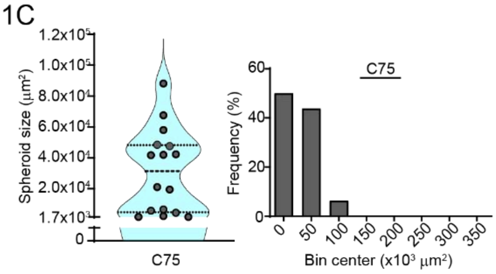

S1D

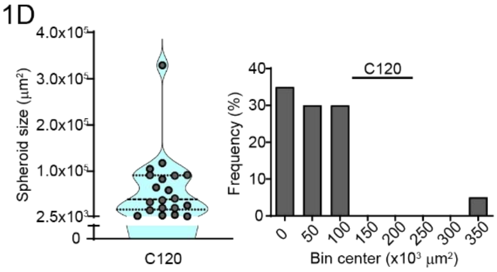

S1E

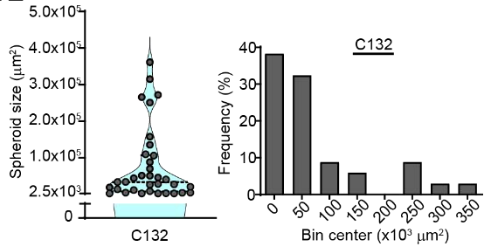

S1F

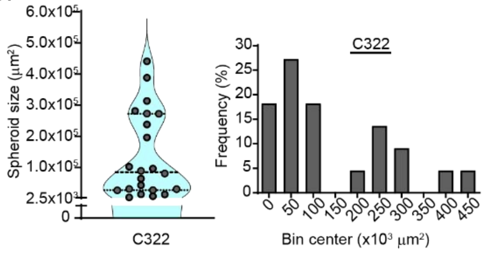

S1G

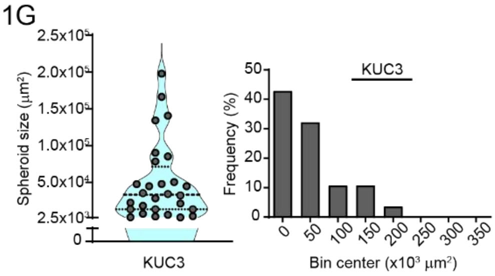

S1H

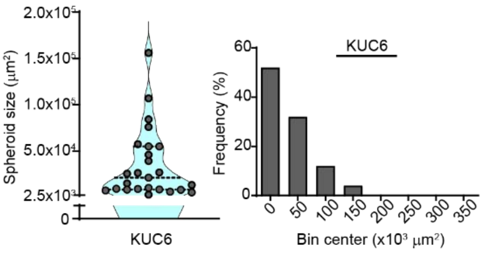

S1I

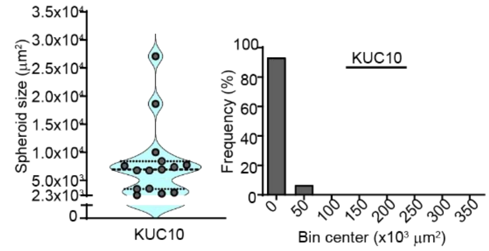

S1J

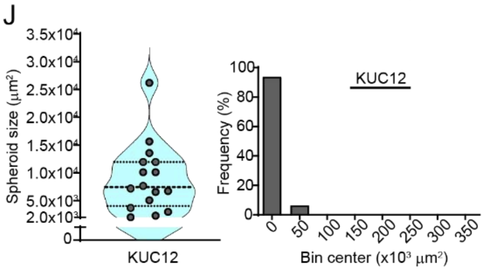

S1K

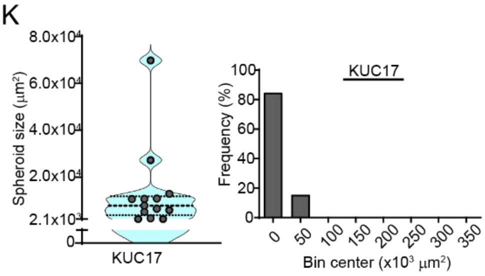

Figure S1. Images of non-growing spheroids and the SSFG assay results of additional CRC organoids, Related to Figure 1.

(**S1A**) Representative phase-contrast images of non-growing spheroids from Figure 1C at the indicated days of culture. Scale bars, 100 μm. (**S1B-S1L**) Violin plots and frequency distribution analysis of the SSFG assay in the indicated CRC organoids lines.

Figure S2

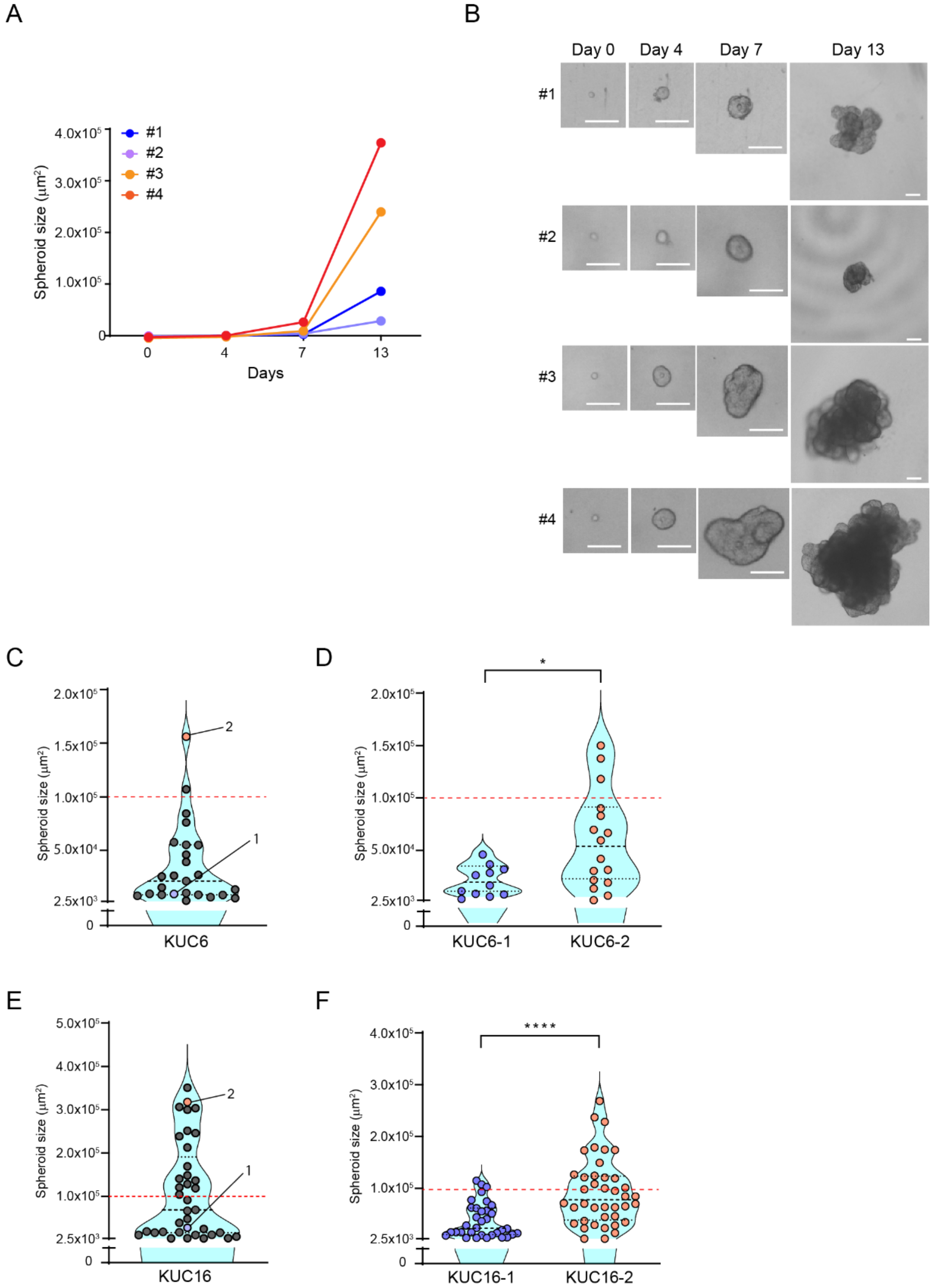
A
B
#1
#2
#3
#4
Spheroid size (μm²)
4.0x10⁵
3.0x10⁵
2.0x10⁵
1.0x10⁵
0
0
4
7
13
Days
Day 0
Day 4
Day 7
Day 13
C
D
E
F
Spheroid size (μm²)
2.0x10⁵
1.5x10⁵
1.0x10⁵
5.0x10⁴
2.5x10³
KUC6
KUC6-1
KUC6-2
*
5.0x10⁵
KUC16
KUC16-1
KUC16-2
****

Figure S2. CRC spheroids exhibit different growth capacities with preserved growth potential, Related to Figure 2.

(**S2A**) Growth curve of the four selected spheroids in Figure 2A. The area of spheroids at different days of culture is indicated. (**S2B**) Time-course images of the selected spheroids in (S2A). Scale bars, 100 μm. (**S2C-S2F**) Violin plots of the SSFG assay for primary CTOSs and the selected clones for KUC6 (S2C, S2D) and KUC16 (S2E, S2F) lines. The selected clones, KUC6-1 and -2, and KUC16-1 and -2, are indicated in S2C and S2E, respectively. The data of the SSFG assay were tested by the Mann–Whitney U test. *, $P < 0.05$; ****, $P < 0.0001$.

Figure S3

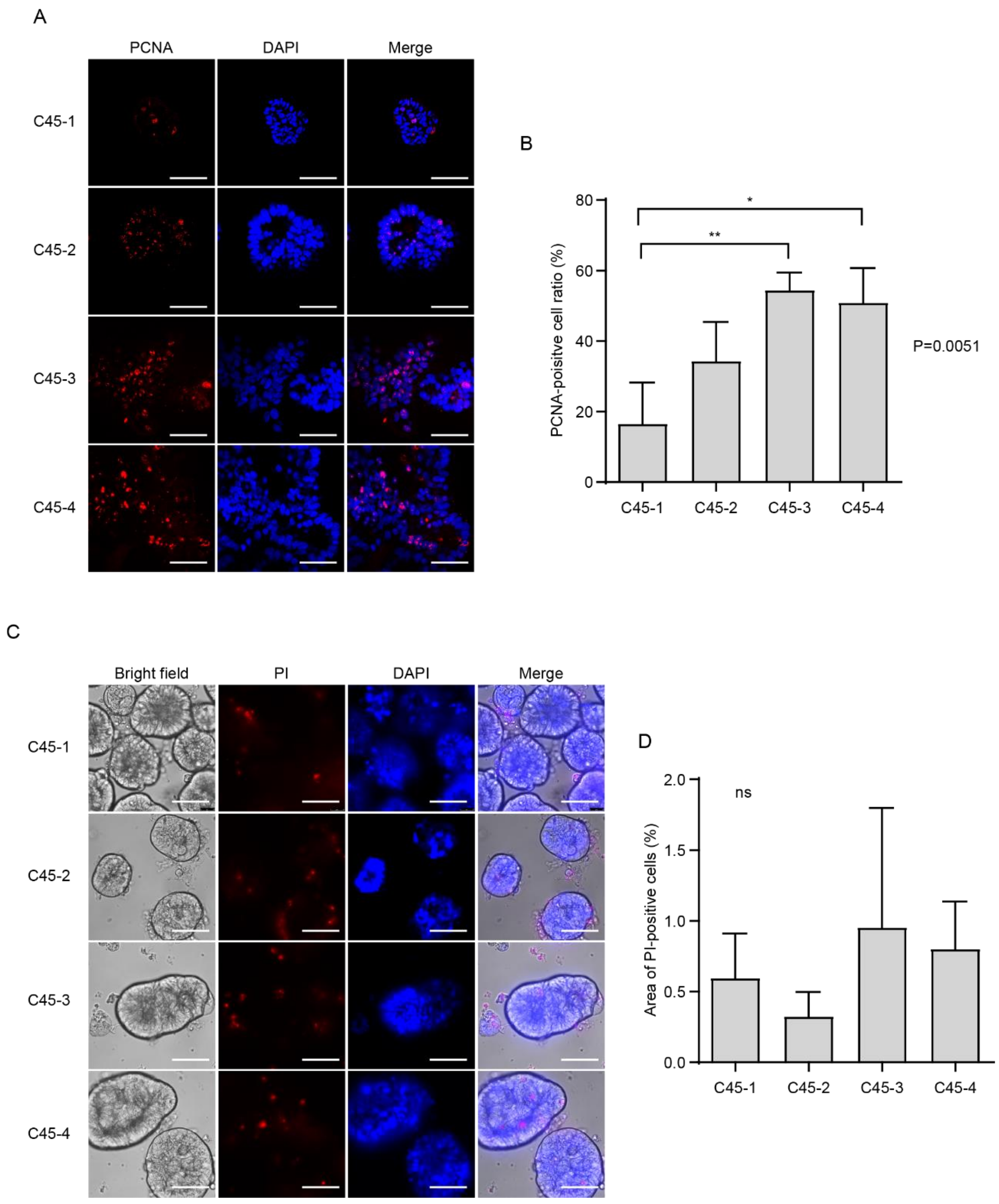

A
PCNA
DAPI
Merge
C45-1
C45-2
C45-3
C45-4
B
PCNA-poisitve cell ratio (%)
80
60
40
20
0
*
**
P=0.0051
C45-1
C45-2
C45-3
C45-4
C
Bright field
PI
DAPI
Merge
C45-1
C45-2
C45-3
C45-4
D
Area of PI-positive cells (%)
2.0
1.5
1.0
0.5
0.0
ns
C45-1
C45-2
C45-3
C45-4

Figure S3. Higher proliferative state, rather than viability, of the cells in the large spheroid-forming clones, Related to Figure 2.

(**S3A**) Representative images of the tumor spheroids stained for PCNA (red) and DAPI (blue) for the indicated C45 clones. (**S3B**) Quantitative analysis of (S3A). The ratio of PCNA-positive cells was calculated and shown as a percentage. Total nuclei number = 226-677. (**S3C**) Representative images of the tumor spheroids stained for propidium iodide (PI) (red) and Hoechst 33342 (blue) for the indicated C45 clones. (**S3D**) Quantitative analysis of (S3C). The area of PI-positive cells was calculated, normalized per total nuclei area, and shown as a percentage. Scale bars, 100 μm. The data were tested by one-way ANOVA, followed by Tukey's test. *, $P < 0.05$; **, $P < 0.01$.

Figure S4

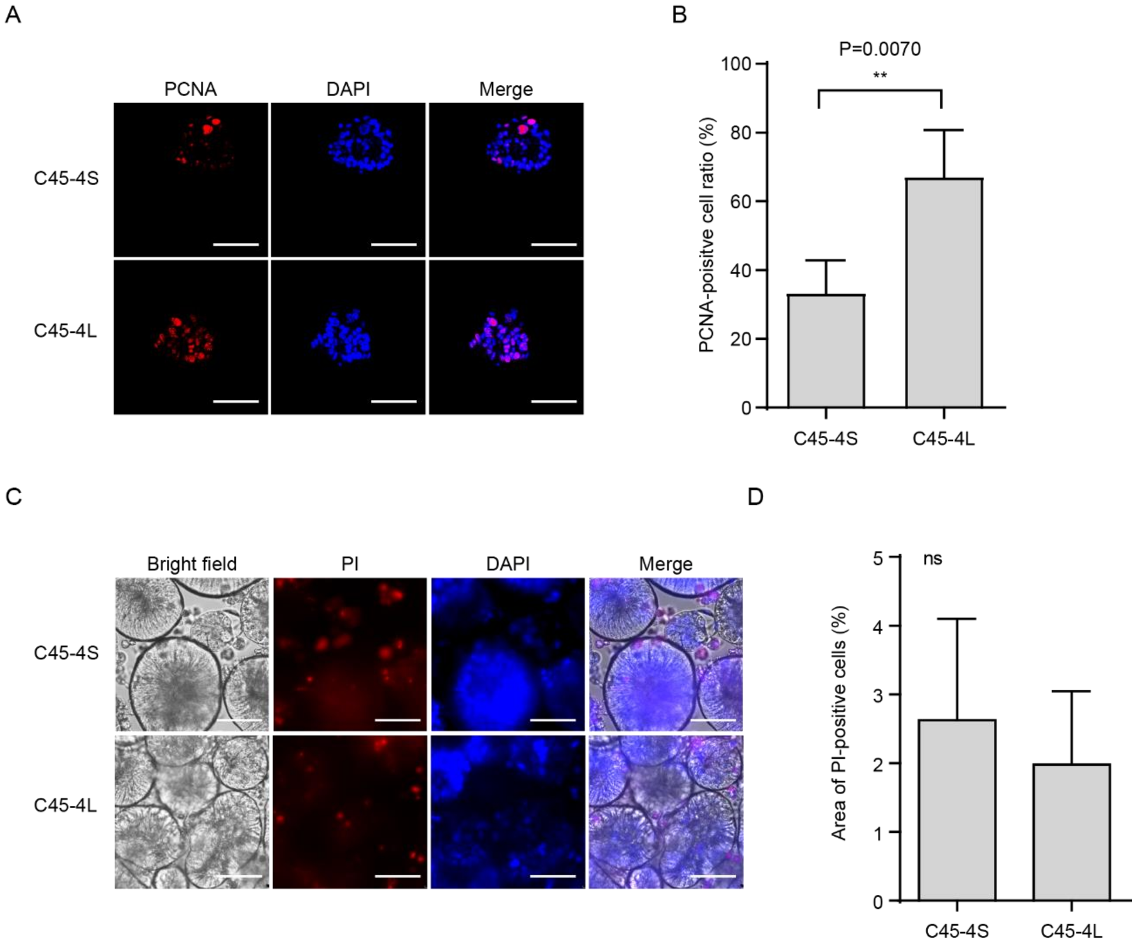

Figure S4. Higher proliferative state, rather than viability, of the cells in the large spheroid subclone C45-4L, Related to Figure 3.

(**S4A**) Representative images of the tumor spheroids stained for PCNA (red) and DAPI (blue) for the indicated C45 sub-clones. (**S4B**) Quantitative analysis of (S4A). The ratio of PCNA-positive cells was calculated and shown as a percentage. Total nuclei number = 173-213. (**S4C**) Representative images of the tumor spheroids stained for propidium iodide (PI) (red) and Hoechst 33342 (blue) for the indicated C45 sub-clones. (**S4D**) Quantitative analysis of (S4C). The area of PI-positive cells was calculated, normalized per total nuclei area, and shown as a percentage. Scale bars, 100 μm. The data were tested by Student t-test. **, P < 0.01. Ns, not statistically significant.

Figure S5

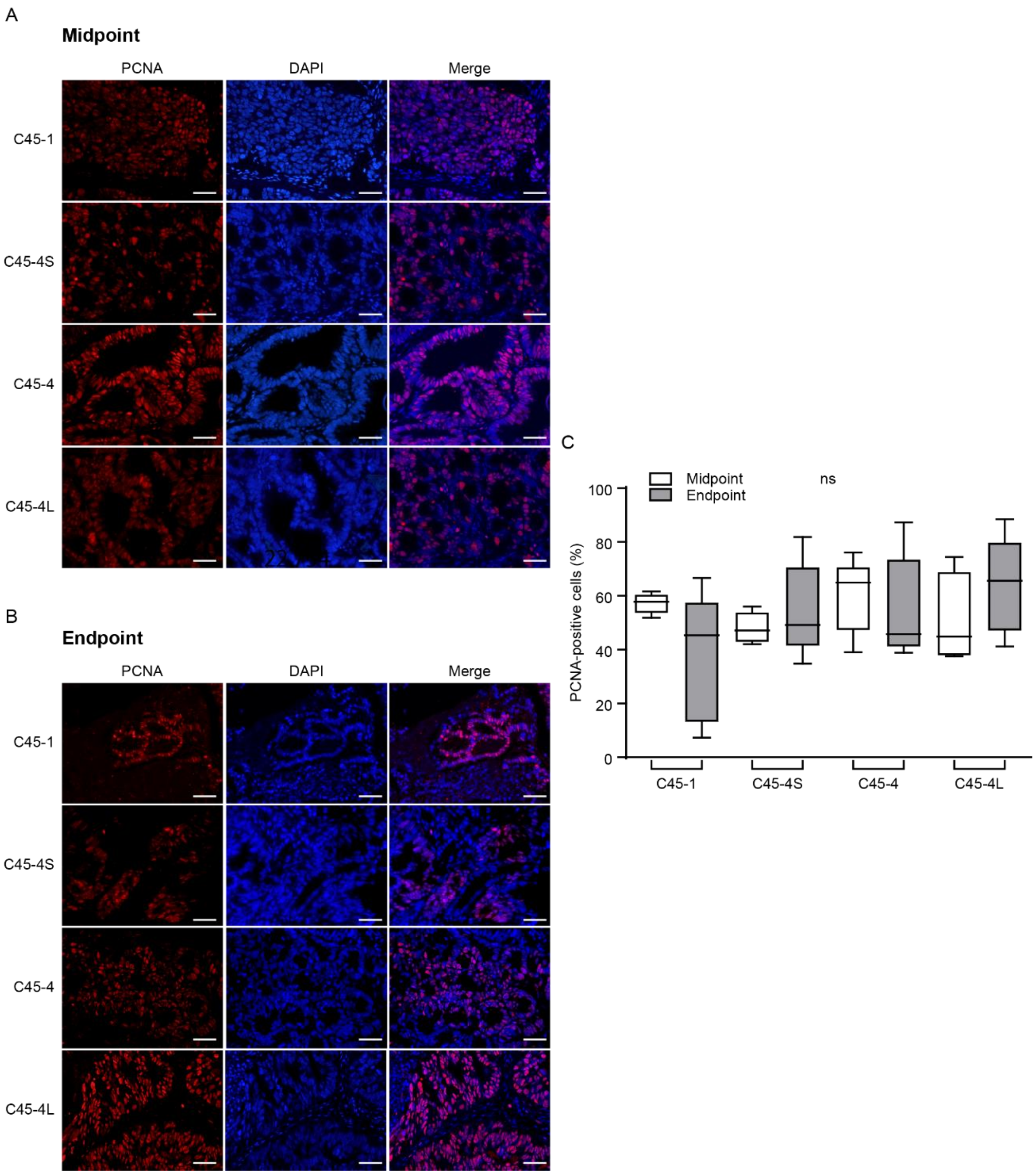
A
Midpoint
PCNA
DAPI
Merge
C45-1
C45-4S
C45-4
C45-4L
B
Endpoint
PCNA
DAPI
Merge
C45-1
C45-4S
C45-4
C45-4L
C
Midpoint
Endpoint
ns
PCNA-positive cells (%)
100
80
60
40
20
0
C45-1
C45-4S
C45-4
C45-4L

Figure S5. S-cells are capable of restarting the cell cycle in vivo, Related to Figure 3.

(**S5A, S5B**) Representative images of the organoid-derived xenograft tumors stained for PCNA (red) and DAPI (blue) at two different time points: a “midpoint” (S5A) and an “endpoint” (S5B) for the indicated C45 clones (C45-1 and C45-4) or subclones (C45-4S and C45-4L). Scale bars, 100 μm. (**S5C**) Quantitative analysis of (S5A and S5B). The ratio of PCNA-positive cells was calculated and shown as a percentage. Total nuclei number =2276-4103. The data were tested by one-way ANOVA, followed by Tukey’s test. Ns, not statistically significant.

Figure S6

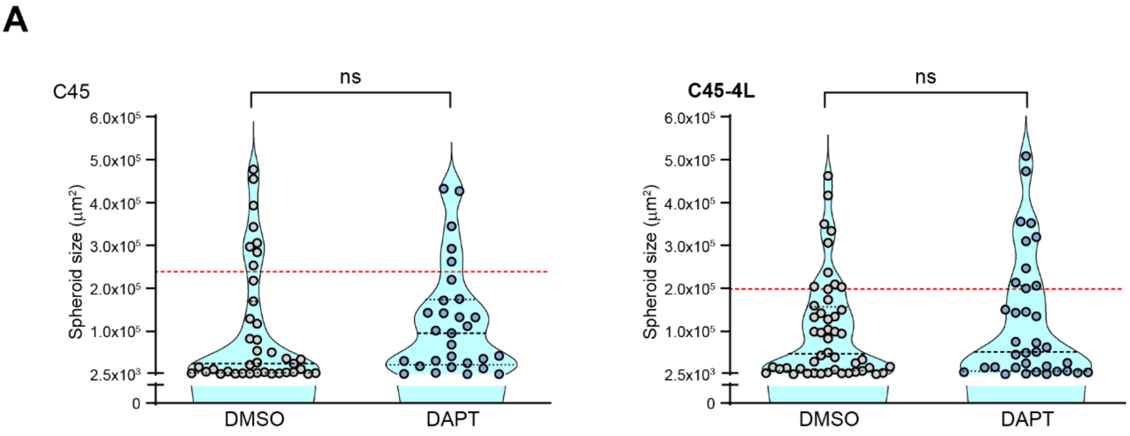
A
C45
ns
6.0x10⁵
5.0x10⁵
4.0x10⁵
3.0x10⁵
2.0x10⁵
1.0x10⁵
2.5x10³
0
Spheroid size (μm²)
DMSO
DAPT
C45-4L
ns
DMSO
DAPT

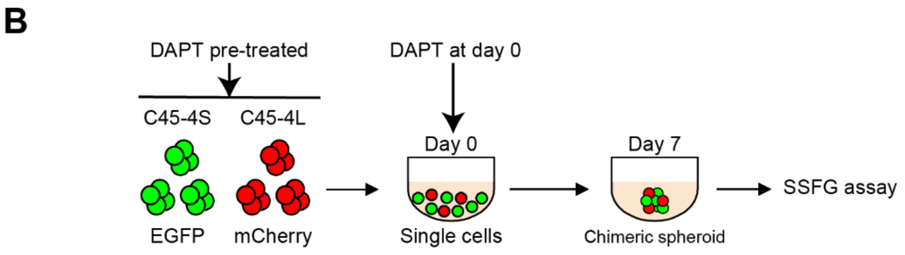
B
DAPT pre-treated
DAPT at day 0
C45-4S
C45-4L
Day 0
Day 7
SSFG assay
EGFP
mCherry
Single cells
Chimeric spheroid

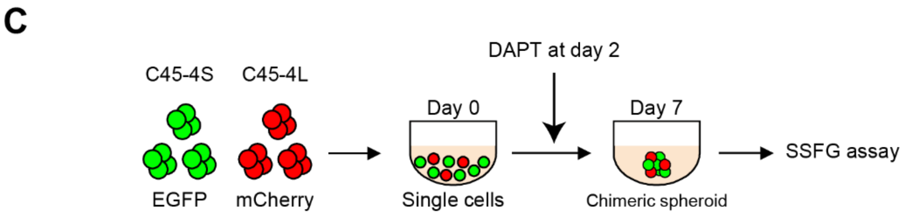
C
DAPT at day 2
C45-4S
C45-4L
Day 0
Day 7
SSFG assay
EGFP
mCherry
Single cells
Chimeric spheroid

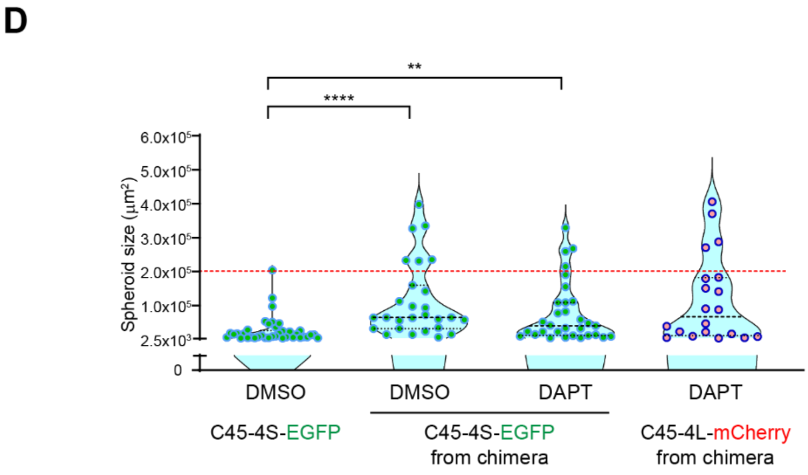
D
**
****
6.0x10⁵
5.0x10⁵
4.0x10⁵
3.0x10⁵
2.0x10⁵
1.0x10⁵
2.5x10³
0
Spheroid size (μm²)
DMSO
DMSO
DAPT
DAPT
C45-4S-EGFP
C45-4S-EGFP from chimera
C45-4L-mCherry from chimera

Figure S6. Inhibition of the Notch signaling in the chimeric spheroids Related to Figure 5.

(**S6A**) Violin plots of the SSFG assay comparing the DMSO-treated with the DAPT-treated parent C45 (left) and C45-4L (right). (**S6B, S6C**) Schematic overview of the chimeric spheroid experiments. The timings of the DAPT treatment are shown. mCherry-labeled C45-4L (red circles) and EGFP-labeled C45-4S (green circles) cells. (**S6D**) Violin plots of the SSFG assay under the same setting with Figure 5I, except for the timing of the DAPT treatment. The data of the SSFG assay were tested by the Mann-Whitney test. **, $P < 0.01$; ****, $P < 0.0001$; ns, not statistically significant.

Figure S7

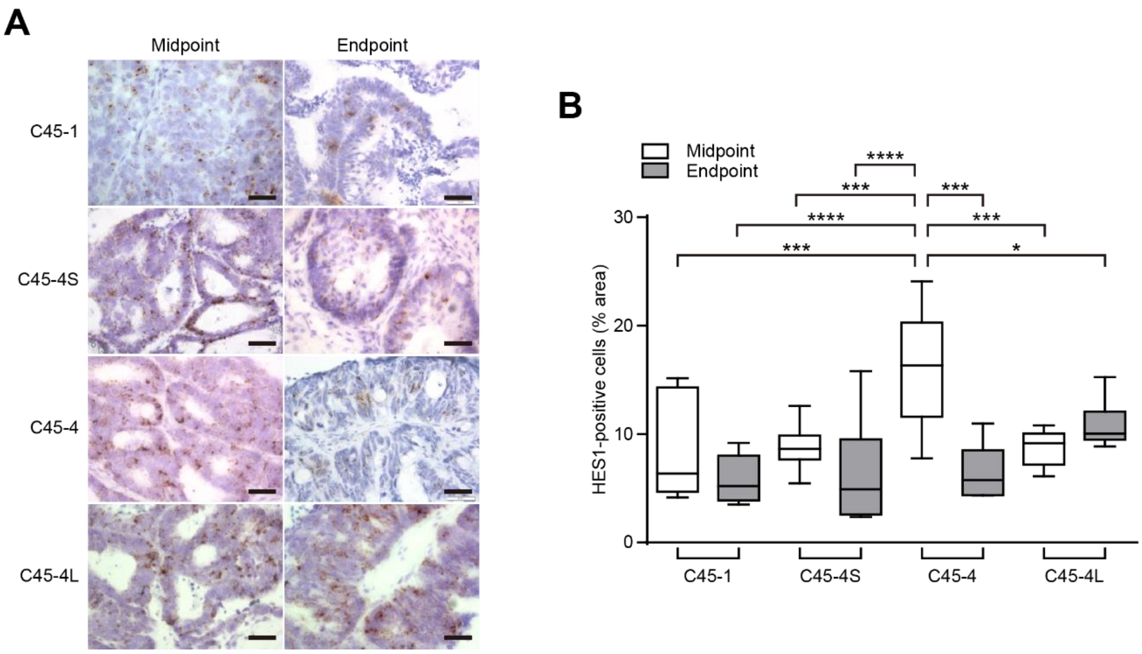

Figure S7. S-cells activate Notch signaling in vivo, Related to Figure 5.

(**S7A**) Representative images of the HES1 in situ hybridization in the organoid-derived tumor xenografts at two different time points; a “midpoint” and an “endpoint” for the indicated C45 clones (C45-1 and C45-4) or subclones (C45-4S and C45-4L). Scale bars, 50 µm. (**S7B**) Quantitative analysis of (S7A). The area of HES1-positive cells was calculated, normalized per total nuclei area, and shown as a percentage. The data were tested by one-way ANOVA, followed by Tukey’s test. *, $P < 0.05$; ***, $P < 0.001$; ****, $P < 0.0001$.

Figure S8

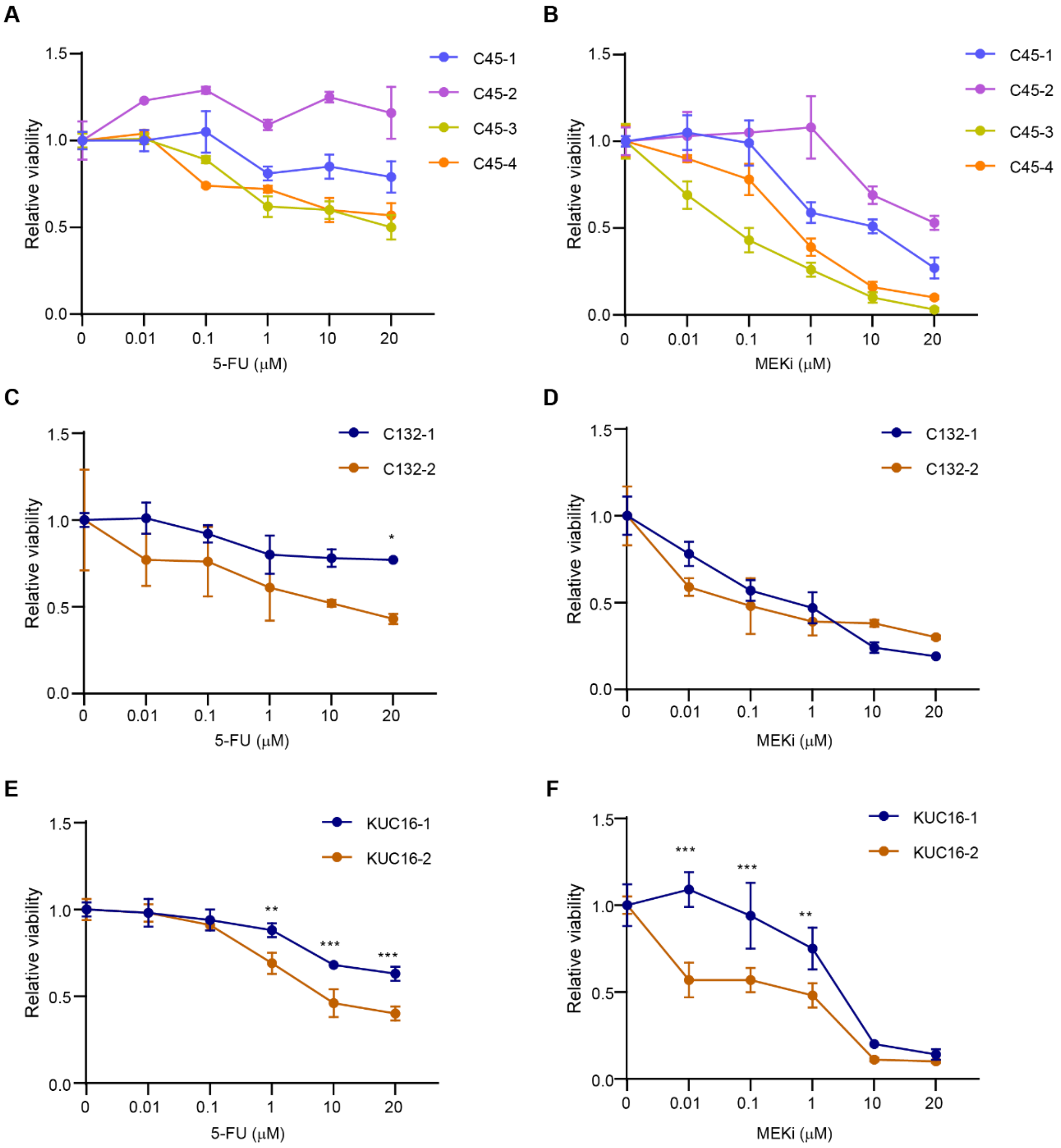

Figure S8. S-cells are the drug-resistant fraction, Related to Figure 6.

(**S8A-S8F**) Dose-dependent curves of the indicated C45 (S8A, S8B), C132 (S8C, S8D), and KUC16 (S8E, S8F) clones treated with 5-FU (left) and MEKi, a MEK inhibitor (right), as evaluated by an ATP assay. The mean ± SD is shown, tested by two-way ANOVA, followed by Tukey's test. *, $P < 0.05$; **, $P < 0.01$; and ***, $P < 0.001$.

Figure S9

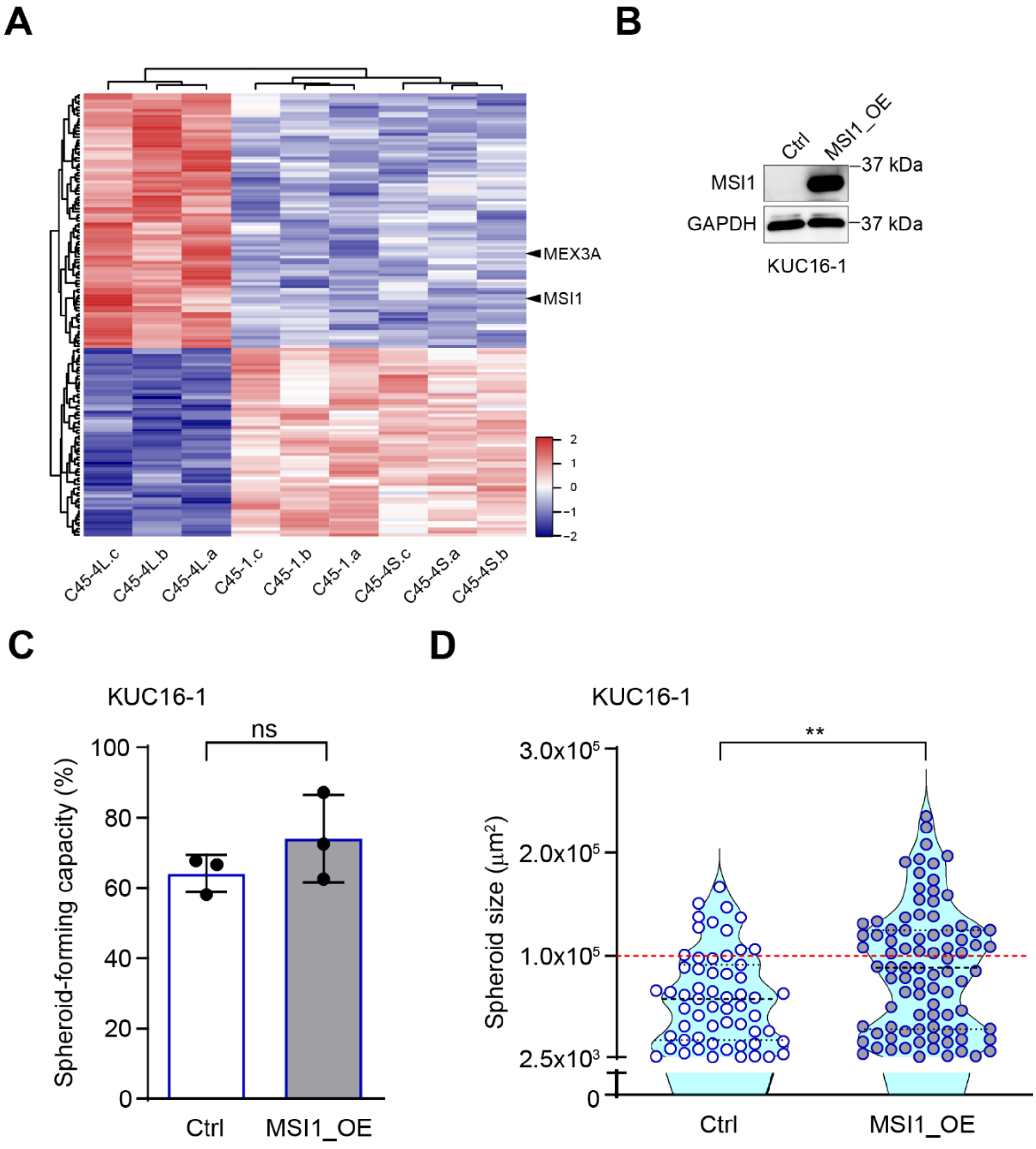
A
B
C
D
MEX3A
MSI1
C45-4L.c
C45-4L.b
C45-4L.a
C45-1.c
C45-1.b
C45-1.a
C45-4S.c
C45-4S.a
C45-4S.b
Ctrl
MSI1_OE
MSI1
GAPDH
37 kDa
37 kDa
KUC16-1
KUC16-1
ns
Spheroid-forming capacity (%)
Ctrl
MSI1_OE
KUC16-1
**
Spheroid size (μm2)
Ctrl
MSI1_OE

Figure S9. The S-cell clone KUC16-1 showed D-pattern by forced overexpression of MSI1, Related to Figure 7.

(**A**) Heatmap and clustering analyses. The probe sets were selected with a relatively high intensity: top 40% of mean expression values, and FDR < 0.1 in the comparison of C43-4L with both the C45-1 and C45-4S lines. MSI1 and MEX3A are indicated. (**B**)Western blot analyses of MSI1 in KUC16-1 spheroids infected with a lentivirus that constitutively expresses MSI1 (MSI1_OE) or with the corresponding empty vector (Ctrl). (**C**, **D**) Spheroid-forming capacity (C) and violin plot of the SSFG assay (D) for the KUC16-1 subclone, comparing the control and the MSI1 overexpressing cells. The data of the spheroid-forming capacity were tested by one-way ANOVA, followed by Tukey's test, and those of the SSFG assay were tested by the Mann-Whitney test. **, P < 0.01; ns, not statistically significant.

Supplementary Table S1. Clinical information of the CTOS lines and the primary tumors, Related to Figure 1.

			Surgical findings							Pathological findings					
Sample ID	Age	Sex	Location	Type	Size (mm)	P	N	M	H	Histology		Depth	INF	ly	v
C45	60s	M	Ra	2	37x60	0	2	0	2	Mod	NA	A	b	1	1
C48	80s	M	Rab	2	53x70	0	0	0	0	Mod	NA	A	b	1	0
C75	40s	F	Ra	3	53x46	0	2	0	2	Mod	NA	SS	b	1	0
C111	70s	F	Rs	2	28x26	0	2	0	0	Mod	tub2>1	SE	b	1	2
C120	70s	M	T	2	47x45	0	0	0	0	Mod	tub2	SS	b	0	0
C132	70s	F	T	2	45x22	0	3	0	0	Mod	tub2	SS	b	1	3
C322	70s	F	S	3	105x90	0	0	1	0	Muc	muc	SE	b	0	0
KUC3	79	M	RS	2	40*40	0	1	0	0	Mod	tub2	SS	b	0	0
KUC6	56	F	Ra	2	15*13	0	2a	0	0	por	por1>>tub2	MP	b	1c	1a
KUC10	64	F	S	2	25*25	0	0	0	0	well	tub1>muc	SS	b	0	0
KUC12	77	F	T	1	28*25	0	0	0	0	Mod	tub2	SM	b	0	0
KUC16	69	F	A	2	50*35	0	0	0	0	Mod	tub2	SS	b	0	1a
KUC17	88	F	A	2	50*45	0	0	0	0	Mod	tub2	SS	b	0	0
KUC18	66	F	T	2	40*35	0	0	0	0	well	tub1>por	MP	b	0	0

Supplementary Table S2. Summary of SSFG assay results in 14 CRC CTOS lines, Related to Figure 1.

C45 was also evaluated after 20 days of culture. Spheroid-forming capacity, minimum and maximum growth size, and growth range of growing spheroids are indicated.

CTOS line	SFC (%)	Minimum growth size ($x10^3$ μm^2)	Maximum growth size ($x10^3$ μm^2)	Growth range (Fold change)
C45	59	2.7	616	228
C48	19	1.7	56	33
C75	20	1.7	88	52
C111	40	2.6	47	18
C120	47	2.6	329	128
C132	51	2.6	583	228
C322	49	3.7	440	120
KUC3	36	2.7	198	74
KUC6	36	3.2	156	49
KUC10	27	2.3	23	12
KUC12	30	2	26	13
KUC16	59	2.6	375	142
KUC17	38	2.1	70	33
KUC18	33	2.8	98	34

SFC: Spheroid forming capacity

Supplementary Table S3. Mutational status of frequently mutated genes in CRC for the CTOS lines and primary tumors, Related to Figure 1.

Mutations are highlighted in gray.

	APC	TP53	KRAS	BRAF	PIK3CA	SMAD4	SMAD2	FBXW7
C45	p.Glu1374*	p.Gly112Ser	p.Gly12Asp	WT	WT	WT	WT	WT
C48	p.Arg1450*	WT	p.Lys117Asn	WT	p.Glu545Lys	WT	WT	WT
C75	p.Arg499*<br>p.Gln1378*	WT	p.Gly13Asp	WT	WT	WT	WT	WT
C111	p.Arg1114*	p.Pro20Leu<br>p.Pro152Leu	WT	WT	WT	WT	WT	WT
C120	WT	WT	WT	p.Lys601Glu	p.Glu545Gly	WT	WT	WT
C132	p.Gln1338*	WT	WT	WT	p.Thr1052Lys	WT	WT	WT
C322	WT	WT	p.Gly13Asp	WT	WT	WT	p.Ser434*<br>p.Ser464*	p.Arg361*<br>p.Arg399*<br>p.Arg479*
KUC3	p.Val452fs<br>p.Arg1114*	p.Arg342*	WT	WT	WT	WT	WT	WT
KUC6	p.Glu1353*	p.Arg196*	p.Gly12Val	WT	WT	WT	WT	WT
KUC10	p.Asn1167fs<br>c.1409-2A>G<br>splice_acceptor<br>_variant	WT	p.Gly12Val	WT	WT	WT	WT	WT
KUC12	p.Arg216*<br>p.Asp285fs<br>p.Arg405fs	p.Lys132Thr<br>p.Pro72Arg	WT	p.Asn581Ile	WT	WT	WT	WT
KUC16	p.Trp421*	p.Val172Phe	WT	WT	WT	p.Cys123fs	WT	WT
KUC17	p.Thr1556fs	p.Gly245Ser	p.Gly12Asp	WT	WT	p.Tyr131Cys	WT	WT
KUC18	p.Ser16fs<br>p.Met1014fs<br>p.Gln1406fs	p.Glu62fs	WT	WT	WT	p.Gln455*	WT	WT

Supplementary Table S6. Sequences of the PCR primers and sgRNA oligos, Related to STAR★Methods.

primer	MSI1_BamHI-F	5′-GGATCCGCCACCATGGAGACTGACGCGCCC-3′
	MSI1_stopdead_XbaI-R	5′-TCTAGAGTGGTACCCATTGGTGAAGGC-3′
	MSI1-F	5′-CACCAATGGGTACCACTGAA-3′
	MSI1-R	5′-ACTCGTGGTCCTCAGTCAGC-3′
	LGR5-F	5′-AGAATTTGCGAAGCCTTCAA-3′
	LGR5-R	5′-TATTTTGTTCAGGGCCAAGG-3′
	CD133-F	5′-TTGTGGCAAATCACCAGGTA-3′
	CD133-R	5′-TCAGATCTGTGAACGCCTTG-3′
	β-actin-F	5′-CCTGGCACCCAGCACAAT-3′
	β-actin-R	5′-GCCGATCCACACGGAGTACT-3′
sgRNA oligo	MSI1 human sense	5′-CACCGACTCAGTTGGCAGACTACGC-3′
	MSI1 human anti-sense	5′-AAACGCGTAGTCTGCCAACTGAGTC-3′
	NT human sense	5′-CACCGTTCCGCGTTACATAACTTA-3′
	NT human anti-sense	5′-AAACTAAGTTATGTAACGCGGAAC-3′
